# The Use of Patient-Reported Outcome Measures in Paediatric Haematopoietic Stem Cell Transplant: A Systematic Review

**DOI:** 10.3390/children13040491

**Published:** 2026-03-31

**Authors:** Rachel Penny, Samantha Keogh, Jill Shergold, Natalie Bradford

**Affiliations:** 1 Cancer and Palliative Care Outcomes Centre, Centre for Children’s Health Research, Queensland University of Technology, Brisbane, QLD 4101, Australia; 2 Queensland Children’s Hospital, Brisbane, QLD 4101, Australia; 3 Centre for Healthcare Transformation, School of Nursing, Queensland University of Technology, Brisbane, QLD 4059, Australia; 4 Nursing and Midwifery Research Centre, Royal Brisbane and Women’s Hospital, Brisbane, QLD 4029, Australia; 5 Viertel Cancer Research Centre at Cancer Council Queensland, Brisbane, QLD 4006, Australia

**Keywords:** paediatric, child, adolescent, Patient-Reported Outcome Measures (PROMs), symptom assessment, nursing assessment, patient outcome assessment, Haematopoietic Stem Cell Transplant (HSCT)

## Abstract

**Highlights:**

**What are the main findings?**

- PROMs are not routinely used in paediatric Haematopoietic Stem Cell Transplant (HSCT) to inform clinical practice.
- There is limited understanding of patient symptom trajectories and symptom experiences during acute HSCT admission.
- Paediatric HSCT recipients with the highest symptom burden are the least represented in current evidence.

**What are the implications of the main findings?**

- There is a call for the integration of PROMs into routine paediatric HSCT care to establish their efficacy in supporting symptom assessment and management and improving clinical outcomes.
- Continued investigation into symptom trajectories, predictive patient factors, and intervention efficacy across the transplant continuum is essential for advancing symptom-focused care in paediatric HSCT.
- There is a critical need for vigilant nursing advocacy and family partnership to ensure accurate symptom identification and clinical practice guideline-based management.

**Abstract:**

Background/Objectives: Children and adolescents undergoing Haematopoietic Stem Cell Transplantation (HSCT) experience complex symptoms, often under-reported by patients and undetected by clinicians, which cause distress. Patient-Reported Outcome Measures (PROMs) offer a way to capture symptom experiences directly from patients, with the potential of supporting effective symptom assessment and management, yet their routine use in paediatric HSCT remains unclear. This systematic review synthesises evidence on PROMs used during inpatient paediatric HSCT care, examining their role in symptom monitoring and clinical decision-making, and identifying gaps to strengthen person-centred, developmentally appropriate care. Methods: We searched the MEDLINE, CINAHL, Embase, APA PsychINFO, and Cochrane Library in October 2024 for studies published in English between 2014 and 2025 describing the use of PROMs during inpatient paediatric (0–18 years) HSCT admission (up to Day +100 post HSCT). In March 2025, prior to data extraction, we added additional studies published by authors of included studies. Two-stage independent screening and data extraction were conducted, and the Quality Assessment with Diverse Studies (QuADS) tool was used to appraise each study. Narrative syntheses informed by Symptom Management Theory were used to compare PROM use, clinical integration, and reported impacts. Results: Seventeen studies met inclusion criteria, describing 20 PROMs used during paediatric HSCT hospitalisation. PROMs captured a wide range of physical and psychological symptoms, with pain and nausea most frequently reported. While PROMs reportedly improve symptom detection and communication, integration into routine paediatric HSCT clinical care was rare; and only two studies systematically used PROMs data to guide symptom management. Evidence of PROMs-driven improvements in HSCT clinical outcomes was scarce, and longitudinal data on symptom trajectories were limited. Conclusions: PROMs are not routinely used to inform clinical practice in paediatric HSCT, and current evidence provides only a partial understanding of symptom trajectories and lived symptom experiences during the paediatric acute transplant admission. To realise the full potential of PROMs in enhancing symptom assessment and management, systematic PROMs integration into clinical workflows is required, supported by electronic health record integration, clinician training, and longitudinal research designs that capture symptom evolution across the transplant continuum.

## 1. Introduction

Child patients with cancer and other non-malignant conditions undergoing Haematopoietic Stem Cell Transplantation (HSCT) face a myriad of challenges, including complex symptomatology that can disrupt their daily lives and significantly impact their quality of life [1,2]. While some symptoms are anticipated, others may arise unexpectedly and go unreported by patients and undetected by clinicians, leading to inadequate management and long-term repercussions [3].

Symptom management in this population is inherently complex. It is shaped not only by the physiological burden of treatment but also by the child’s or adolescent’s capacity to recognise and communicate their symptoms, and by the interplay between their experience and that of their parents or caregivers [4]. Effective symptom management begins with symptom recognition, where accurate identification and understanding of a patient’s experience provides the foundation for the appropriate clinical response [5].

Patient-Reported Outcome Measures (PROMs) are used in research and clinical practice to capture outcomes as directly experienced from the patient’s perspective. They can be used as symptom screening tools to include the voice of the child or young person in symptom assessment [6]. PROMS are especially valuable for detecting and assessing symptoms that are experienced by, and thus best known to, the patient and may fluctuate in ways not otherwise visible in clinical charts or records. These tools typically screen for both physical and psychological symptoms and allow the patient to rate the presence and severity of symptoms through age-appropriate questions and scales [7]. Depending on the developmental stage or clinical condition, PROMs may be completed by the child or adolescent themselves, by a parent or carer acting as a proxy respondent, or through co-completion by both the child and parent [8,9].

The use of PROMs in adult HSCT demonstrates feasibility and acceptability and several clinical benefits, including reduced peak symptom burden, improved health-related quality of life (HRQoL), and improved patient–clinician communication and mental functioning [10,11,12]. Other studies conducted in post-transplant care and general paediatric oncology contexts have also demonstrated benefits including enhanced patient–clinician communication and more responsive and person-centred care planning [13,14]. However, the application of PROMs in paediatric contexts presents unique challenges, particularly the need for developmentally appropriate formats, the potential for shared or proxy completion, and the interpretive complexity that can arise when children’s self-reports diverge from parent or clinician assessments [7,8]. Despite the high burden of symptoms in paediatric HSCT, little is understood about the use of PROMs in this context.

Therefore, the aim of this systematic review is to identify the PROMs used during inpatient admission for paediatric HSCT and to evaluate how these tools contribute to the understanding and management of symptom experiences in this unique and vulnerable patient population.

## 2. Materials and Methods

This review was conducted in accordance with established systematic review methodology, following guidance from the Cochrane Handbook for Systematic Reviews of Interventions [15]. A detailed protocol outlining the objectives, eligibility criteria, and planned methods was prospectively registered with PROSPERO (registration number: CRD420250572124) [16]. Unpublished studies, editorials, letters and conference proceedings were not sought; however, reference lists of eligible studies were manually searched.

Studies were eligible for inclusion if they reported on children and young people (0–18 years), with any malignant or non-malignant condition, receiving HSCT treatment. Specifically, the inclusion of patients that were less than or equal to 100 days post HSCT were sought. Studies had to include the use of PROMs or a standardised assessment tool reported by the patient (or their proxy) in the inpatient setting. There were no restrictions on the types of study design eligible for inclusion.

Studies were excluded if they only reported on adults aged ≥18 years or reported on any treatments other than HSCT. Studies where patients were receiving care in the outpatient setting, where patients were discharged, and those at more than 100 days post HSCT were also excluded.

The search strategy was developed in consultation with a university librarian and was designed to capture literature at the intersection of symptom assessment and paediatric HSCT, with a focus on symptom screening during inpatient admission. To ensure comprehensiveness and validity, the strategy was tested to confirm its ability to retrieve a sample of known eligible studies [3,17,18]. The university librarian subsequently reviewed the search strategy using the Peer Review of Electronic Search Strategies (PRESS) 2015 evidence-based checklist [19]. The search was conducted in five databases: MEDLINE, CINAHL, Embase, APA PsychINFO, and Cochrane Library and limited to English publications and publications between 2014 and 2025 to reflect contemporary research and practice. A combination of free text terms, synonyms and MeSH terms with Boolean operators were used to combine key concepts of patient, assessment, and HSCT. For transparency and reproducibility, the full search strategy is provided in Table A1 of Appendix A.

All studies were uploaded to the Covidence application (2026 web-based SaaS; Veritas Health Innovation Ltd, Melbourne, VIC, Australia), for screening. Duplicates were removed and the eligibility criteria was applied to all titles, abstracts and full text during screening. Given the purpose of this review and the heterogeneity of included study designs, a pragmatic approach to study selection and data handling was adopted. Title and abstract screening were conducted in pairs using a two-reviewer process. To maintain consistency across screening decisions, one primary reviewer (RP) participated in both reviewer pairs, while the second reviewer differed between pairs. Each pair independently screened titles and abstracts against the predetermined eligibility criteria.

Inter-rater reliability was evaluated using Cohen’s kappa (κ), which adjusts for chance agreement. Inter-rater reliability was assessed at title/abstract screening and full-text screening using Cohen’s kappa (κ) statistic, calculated to evaluate the level of agreement beyond chance, and interpreted using the widely cited Landis and Koch scale [20]. This approach aligns with best practice in systematic review methodology and PRISMA guidance, which recommends kappa because it adjusts for chance agreement. Percent agreement was also calculated to provide descriptive context for κ values.

Discrepancies between reviewers were resolved through discussion, and if consensus could not be reached, a third reviewer was consulted to independently check the studies. All votes were blinded during the resolution of any conflicts. This process ensured rigour, transparency, and reproducibility throughout the study selection.

Due to the substantial volume of data extracted across diverse study types, data extraction was undertaken by one reviewer (RP) and subsequently checked for accuracy and completeness by a second reviewer. Discrepancies were resolved through discussion and, where they were unable to be resolved, a third person was consulted. Data extraction was performed using the Covidence application and exported for analysis.

The following data were extracted from included articles:

- Type of study/study design and methodology
- Participant demographics/baseline characteristics including age and location of care
- Type of PROMs used—(e.g., validated, disease or age-specific tool)
- Number and timepoints of data collection via PROMs
- Outcome of PROM use (e.g., for research purposes or to inform clinical care)
- The role/discipline stream of the healthcare professional receiving the PROMs data.
- PROMs scores (where applicable)
- Data describing identification and communication of symptoms.
- Barriers and enablers to the use of PROMs/standardised tools identified by patient, parents/carers, and healthcare professionals.
- Referrals made/impacts on care.

This dual-step verification process was also applied during the quality assessment stage which was conducted using the Quality Assessment with Diverse Studies (QuADS) tool to provide a descriptive assessment of methodological rigour across the included studies. The QuADS tool demonstrates strong reliability and ease of use for application to systematic reviews with mixed or multi-methods health services research [21]. The QuADS tool assesses quality of the body of evidence against 13 items. Assessment of each criterion was made on a scale of 1–4 (0—not at all; 4—completely). Quality assessment was performed independently by one person, using the QuADS tool. A selection of assessments was checked by a second reviewer and any discrepancies in judgement were resolved through discussion. QuADS scores were used to characterise common strengths and limitations of the evidence base but did not inform study inclusion, exclusion, or weighting in the synthesis.

Given the heterogeneity of populations, outcomes, and study designs, findings were synthesised narratively to compare the types of PROMs used, the domains assessed, and their integration into clinical care. The synthesis was informed by Symptom Management Theory (SMT)**,** which provided a conceptual framework and lens for examining how symptoms were perceived, communicated, and managed [22]. Specifically, the components of the SMT, symptom experience, symptom management strategies, and outcomes were used to structure the analysis and interpretation of included studies. Qualitative and descriptive findings were organised around how PROMs captured the symptom experiences of children and young people, how these experiences informed clinical responses (symptom management), and what outcomes were reported. This approach supports a theory-informed understanding of the role of PROMs in paediatric HSCT and helped identify conceptual and practical gaps in the current evidence base. No subgroup analyses were performed.

## 3. Results

### 3.1. Overview of Included Studies

This systematic review was conducted in compliance with the Preferred Reporting Items for Systematic reviews and Meta-Analyses (PRISMA) 2020 statement which provides guidance for reporting systematic reviews [23,24]. The full PRISMA 2020 and PRISMA 2020 for abstracts checklists for this review can be found in the Supplementary Materials.

#### 3.1.1. Study Selection

A total of 1972 potentially eligible records were identified through database searching. After removing 216 duplicates, 1756 records remained for title and abstract screening. Of these, 1691 were excluded based on the predefined eligibility criteria. The full text of the remaining 65 articles was assessed for eligibility. Forty-eight articles were excluded, with 17 studies included in the final review. Inter-rater reliability statistics demonstrated acceptable agreement across screening stages. For title and abstract screening, percent agreement ranged from 72.5% to 95.7%, with κ values between 0.353 and 0.442, indicating fair to moderate agreement. For full-text screening, percent agreement ranged from 75% to 77%, with κ values between 0.448 and 0.522, reflecting moderate agreement between reviewer pairs. All discrepancies were resolved through discussion, and a third reviewer was consulted when consensus could not be reached.

**Figure 1:**
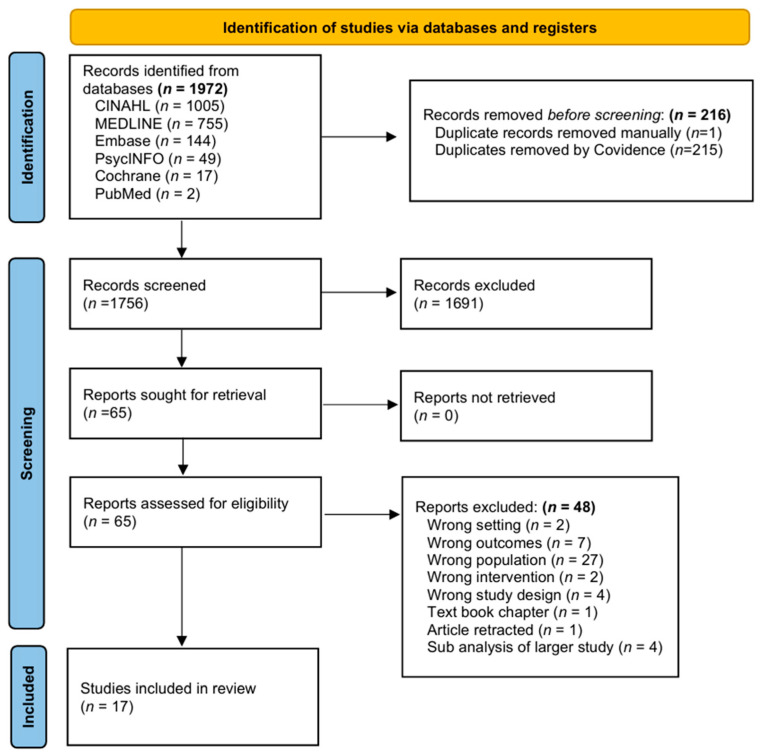
PRISMA flow diagram of article selection.

The results of the study selection can be viewed in Figure 1, PRISMA flow diagram.

#### 3.1.2. Study Characteristics

The seventeen studies were published in 14 different journals; see Table A2 of Appendix B. Characteristics of included studies. Seven studies were conducted in United States of America (USA), four in Canada, three in both USA and Canada, and one study was conducted in Austria, Brazil, and Turkey. The review included eleven prospective cohort studies, five randomised controlled trials and one pilot study. Most (94%) studies included paediatric oncology patients with HSCT participants reported as a subgroup. Participant numbers in individual studies ranged from four to 502 with a total of 2446 paediatric participants represented across all studies, 490 (20%) of whom were paediatric HSCT recipients. Where reported, male participants represented between 19.2 and 61.4 percent. Malignant diseases represented in the paediatric studies included children with leukaemia, making up the highest proportion, ranging from 42% to 66% of study participants and representative of chid cancer incidence patterns in high-income countries [25]; followed by children with solid cancers, lymphomas and brain tumours. Non-malignant conditions were reported in 14 studies and included aplastic anaemia, myelodysplastic syndrome, sickle cell disease, haemophagocytic lymphohistiocytosis, Wiskott–Aldrich syndrome, immune deficiencies, and metabolic disorders. See Table A2 of Appendix B for complete characteristics of participants in included studies.

Nine studies were multisite, involving between two and nine sites. Two of these studies were conducted exclusively in HSCT settings and the other seven across oncology services, including HSCT participants as a proportion of the total number of participants, as outlined in Table A2 of Appendix B. Seven of the eight single-site studies were conducted exclusively in a HSCT unit with the remaining single-site study capturing both oncology and HSCT participants. The percentage of HSCT participants included in these studies as a proportion of the total number of participants ranged between 6.4 and 33.3 percent. One study [26] included paediatric patients aged up to 17 years as a proportion (13%) of all participants. All other studies exclusively captured paediatric participants. Many of the included studies collected data from oncology and HSCT participants in both the inpatient and outpatient settings. Where paediatric HSCT specific symptom data was not specified in the manuscript, authors were contacted via email [3,9,26,27,28]. No further data was made available for synthesis.

#### 3.1.3. Quality Appraisal

Most studies included were of a moderate to high standard as detailed in Table A3 of Appendix C. The majority (82%) of the included studies were conducted by a consortium of researchers in the USA and Canada with experience in conducting research focused on PROM use in the paediatric oncology setting. Most (98%) studies included in this review had a clear description of the research setting and target population, used appropriate data collection tools and were appropriately designed to address the stated research aims. Few (24%) studies described specific theories or concepts that informed each element of the study design. Across most (88%) studies, there was a notable lack of reported evidence that research stakeholders had been considered in the research design or study conduct.

### 3.2. Overview of PROMs

#### 3.2.1. Types of PROMs

There were 20 different PROMs described in the included studies. Full PROMs details and implementation characteristics can be found in Table S1 of Supplemental Materials. Characteristics of PROMs used in included studies. Sixteen of the PROMs were validated. Two studies used the Memorial Symptom Assessment Scale (MSAS) which is validated for use in patients of any age with cancer [29,30]. These same authors in separate studies used the Paediatric Quality of Life and evaluation of symptoms study (PQ-MSAS), an adaptation of the MSAS for children aged 7–12 years and 13–18 years [18,31]. Three studies used unvalidated PROMs [3,28,31], two of which described novel PROMs and their validation processes [3,28]. Two additional studies failed to report whether the PROM being used was validated or not [17,32]. Both of these studies reported on the use of a mobile platform or software system designed to deliver and record PROMs rather than a single PROMs measure [17,32]. These electronic platforms as such are not validated PROMs; however, they deliver symptom questionnaires and have been shown to be feasible with evidence of validity and reliability in the paediatric oncology clinical context [17,32,33].

#### 3.2.2. Domains Assessed

**Figure 2:**
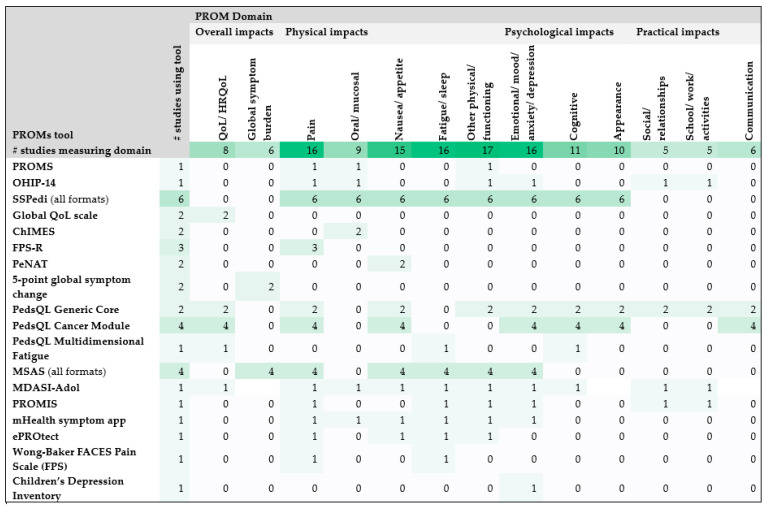
Number of studies that measured each symptom domain and the PROMs used.

The PROMs used in the included studies assessed a broad range of physical, psychological, social, and quality of life domains, reflecting the complexity of the symptomatology experienced by patients during HSCT [3,9,17,18,27,28,29,30,31,32,34,35,36,37,38,39]. As seen in Figure 2, the most common physical domains assessed were pain, fatigue and nausea/appetite, whilst mood, including anxiety and depression, were the most common psychological domains assessed.

##### Global Quality of Life

Global Quality of life was measured in eight studies using one of three different PROMs, the Peds QL 4.0, Peds QL Cancer Module, and the Global Quality of Life (GQoL) visual categorical scale [3,18,27,28,29,30,31,35,36].

##### Physical

Eight PROMs assessed an individual physical symptom domain, such as depression, fatigue, mucositis, nausea, or pain [3,26,28,35,39], with one PROM, the Global Symptom Change Scale, assessing the overall change in symptoms [3,28].

Pain was the most common physical symptom domain captured across all studies. It was assessed by individual symptom PROMs such as the Wong–Baker FACES Pain Rating Scale, the Face Pain Scale–Revised (FPS-R), and the Patient-Reported Oral Mucositis Symptom (PROMS) scale [3,26,28,35,39]. Pain was also included and assessed within the physical domain in all multidimensional PROMs. Three PROMs, the OHIP-14, CHIMES and PROMS, were used in studies to assess mucositis. CHIMES was used by Dupuis et al. [3] and Tomlinson et al. [28] to perform construct validation of the SSPedi and Co-SSPedi tools respectively, whilst PROMS was used by Bezinelli et al. [26] to measure the effectiveness of low-level laser therapy on oral mucositis in HSCT patients.

Nausea was a domain reported using the Paediatric Nausea Assessment Tool (PeNAT) as an individual domain and was included in many of the multidimensional PROMs including the PedsQL CM™, MSAS, MDASI-Adol, MSAS 10-18, PQ-MSAS, SSPedi, Co-SSPedi, and ePROtect [3,9,17,18,27,28,29,30,31,34,35,36,38,39].

##### Psychological

Psychological domains such as mood, distress, discomfort, disability, feeling disappointed/sad, scared/worried, cranky/angry, procedural/treatment anxiety, and emotional functioning were assessed in the studies using multidimensional PROMs, and Yildiz et al. [39] also used a single-symptom domain PROM—the Children’s Depression Inventory.

#### 3.2.3. Implementation of PROMs

##### Target Respondent

Most PROMs included in the studies were designed or adapted for use by children and young people, using strategies such as age-appropriate scales and pictural representations of symptoms or distress. These can be completed through a self-report by children; however, in the case of younger children, they have the option of parent proxy-reporting. Some PROMs are validated for patient self-report at certain developmental age ranges, e.g., SSPedi (8–18 years), PROMIS Paediatric (8–17 years), PROMS (8–18 years), MSAS 10-18, and MDASI-Adol (13–17 years), and some for co-completion, e.g., Co-SSPedi and the 5-point global symptom change scale [3,9,26,28,37]. Overall, co-completion was described in two studies [9,28], and the remaining studies described patient self-report with the option of parent proxy-reporting for younger children.

##### Frequency and Mode of Administration

Electronic completion of PROMs occurred in ten studies, through a handheld smart phone, tablet or other electronic device. In three studies, PROMs were completed in paper form [18,27,36] and in one study, participants were able to complete the PROMs in either paper or electronic form [30]. Four studies did not report the method of PROM completion [26,29,37,39].

Symptoms were captured by PROMs in a variety of ways, either before and after transplant, daily, weekly, or every 30 days, with only two studies capturing daily symptom data in the first 30 days of transplant [17,32]. Of the studies that were conducted exclusively in the inpatient HSCT setting, nine measured PROM data at baseline and post discharge with no longitudinal data collected during hospital admission. The timepoints and frequency of PROM assessment varied for each study during hospital admission, with no consistency demonstrated between studies. There were only two studies that captured daily longitudinal HSCT PROM data [17,32]. Hetzer et al. [17] collected daily PROM data throughout the acute phase of the transplant, up to 22 days post reinfusion of cells. Ford et al. [32] collected daily PROM data for up to 120 days; however, this was a pilot study limited by a small sample size of only four participants.

### 3.3. Outcomes of PROMs

#### 3.3.1. Symptom Experience

##### Physical Symptom Burden

Nausea, fatigue, mucositis, and pain were consistently identified as prominent physical symptoms experienced during HSCT [17,31]. In studies that examined symptom experience between HSCT and non-HSCT patients, the severity of all symptoms was increased in patients following HSCT. For example, nausea was one of the most frequently reported symptoms across multiple studies, with distress scores for nausea significantly higher among HSCT patients compared with non-HSCT groups [31]. Hetzer et al. [17] identified nausea and appetite loss along with pain, physical functioning, and sleep disturbance as the most burdensome physical symptoms, particularly between Day +4 and Day +12 post HSCT and Rodgers et al. [29] found that vomiting was the most frequently reported symptom at one month post HSCT. Fatigue and sleeping difficulty were also notable [27,31], with Ward et al. [31] noting that HSCT patients consistently reported higher fatigue scores compared with non-HSCT oncology patients. HSCT patients also reported higher symptom severity and distress scores for lack of appetite as well as frequency and distress for diarrhoea than non-HSCT patients [31].

##### Psychological and Emotional Symptom Burden

Psychological distress including sadness, worry, and treatment-related anxiety was reported across several studies [27,31,32]. Two studies identified ‘feeling sad’ as the most prevalent psychological symptom [27,31]; however, Ward et al. [31] found no difference in psychological symptoms between transplant and non-transplant children. Emotional symptoms, where reported, were associated with significant reductions in health-related quality of life HRQoL [27,29,32]. Rodgers et al. [29] identified that symptoms of sadness, tiredness, and insomnia were strongly correlated with lower HRQoL during the recovery phase, with this association persisting for several months post HSCT. In their study of symptom distress and mood, Ford et al. [32] found a strong presence of emotional distress and its association with symptom distress and mood—in particular, the associations between feeling worried and sad, the emotional distress caused by feeling worried or angry, and the connection between the severity of feeling tired and feeling worried [32]. Sheikh et al. [27] noted a significantly increased symptom-related interference with general mood and enjoyment of life post-transplant.

##### Symptom Variations Between Child, Parent, and Dyadic Reporting

PROM findings revealed notable discrepancies between child self-report, parent proxy, and dyadic (co-completion) approaches. Tomlinson et al. [9] showed that proxy reports often underestimated the severity of sadness and irritability, while overestimating fatigue. In contrast, in their other study, they found that co-SSPedi scores provided a middle ground, reducing, but not eliminating, discrepancies between self- and proxy reports [28]. Tomlinson et al. [9] went on to test the various approaches to symptom assessment and completion of PROMs using SSPedi. While mean total SSPedi scores did not differ by patient or proxy report, individual symptom scores were significantly different for five symptoms: feeling disappointed or sad, feeling cranky or angry, feeling tired, mouth sores, and changes in taste.

##### Symptom Trajectories

Only two studies described the trajectory of symptoms across the acute transplant phase [17,32], with Hetzer et al. [17] identifying peak burden during the first two weeks post-transplant. Hetzer et al. [17] captured the level of bother a child experienced from the symptom using SSPedi whilst Ford et al. [32] examined the relationships between symptom distress and mood using network analysis.

Where longitudinal data were available, PROMs highlighted distinct trajectories in symptom experience. Hetzer et al. [17] observed that during the acute admission for HSCT, participants experienced an increase in symptom burden from baseline across all PROM scales, with a resolution of almost all symptoms from week 2 onward aligned to engraftment. Hetzer et al. [17] also note that peak symptom burden was experienced from nausea, pain, physical functioning and sleep disturbance between Day +4 and Day +12 of transplant whilst Rodgers et al. [29] report vomiting as the most common symptom at one month post HSCT. Importantly, Rodgers et al. [29] also observed that symptoms experienced at one month post HSCT such as feeling tired, sad, worried, and having insomnia correlated with lower HRQoL scores in the subsequent 5 months post HSCT. Notably, very few studies collected continuous daily or weekly PROM data, significantly limiting understanding of how symptoms evolved over time, particularly during the acute admission for HSCT.

#### 3.3.2. Use in Clinical Practice

##### Integration into Clinical Practice

Of the seventeen studies, only two reported using the PROMs in implementation where data were available for the clinical team to review [17,35]. Dupuis et al. [35] measured scores for 5 consecutive days in a mixed group of participants from both oncology and HSCT settings. The SSPedi reports of the intervention group from Days 1–4 were shared with the bedside nurse, treating team, and placed in the patients’ health record. On Days 1 and 3, emails were sent to the responsible physician if any symptom was “a lot” or “extremely” bothersome. Reports and alerts contained links to clinical practice guidelines. Hetzer et al. [17] used ePROtect on a daily basis throughout the HSCT treatment period, and the scores were reviewed by the treating team before the morning visit, 7 days a week. Any score deviations were discussed immediately and interventions initiated as per standard operating procedures. These two studies were conducted in the last two years and included a total of 39 HSCT patients receiving the PROMs as an intervention to support clinical care.

In two additional research studies, the PROM data was uploaded to the electronic medical record [27,34], but the use of the information to inform clinical practice was not described. All other studies used PROMs to answer a research question, validate an assessment tool, or measure the outcome of an intervention, and did not use PROM reports to inform clinical care.

##### Communication and Care Processes

When PROMs were shared with clinical teams, they enhanced communication between patients, parents, and clinicians. Dupuis et al. [35] identified that symptom screening was associated with significantly more documented interventions for tingly or numb hands or feet, hurt or pain, and problems with thinking or remembering. Hetzer et al. [17] report that daily PROM reviews prompted timely discussions and immediate intervention when symptom deviations were noted. Similarly, Dupuis et al. [35] demonstrate that electronic alerts improved bedside nurse and physician awareness of bothersome symptoms. However, outside these examples, PROM data were rarely described as influencing team communication or care planning.

##### Documentation

Dupuis et al. [35] found that symptom screening was associated with an increase in documentation for some symptom domains (hurt or pain, feeling more or less hungry, and mucositis) and with more documented interventions for constipation. For those symptoms that caused severe bother, screening was associated with more documentation for constipation and more documented intervention for feeling cranky or angry. Overall, symptoms were documented or treated significantly more often with symptom screening [35].

##### Barriers and Enablers of Implementation

Several barriers to the use of PROMs in paediatric HSCT settings were identified. Two studies found that the most prominent challenge was the impact of symptom severity, with children experiencing high treatment-related burden often finding it difficult to complete PROMs consistently or at all [34,38]. This highlights the paradox that those with the greatest symptom burden may be the least represented in outcome data. Forgetting to complete the PROMs was another barrier [34]. In addition, the lack of seamless integration of PROMs into electronic medical record (EMR) systems and clinical workflows limited clinician engagement with PROM data. These issues reflect broader challenges of PROM implementation, in particular the feasibility and sustainability of PROM use in routine care.

In contrast, several enablers were reported that supported greater uptake and acceptability of PROMs. The use of electronic platforms with automated reminders or alarm features was the most common enabler to completing PROMs [34,38]. Cook et al. [34] also found that a reminder from a parent or research associate helped participants remember to complete PROMs. Johnston et al. [36], in contrast, described the challenges of consistent PROM completion during clinical trials and how, despite reminders from clinical research assistance and the addition of a coordinator, sustained improvements in completion rates were not evident for the duration of the clinical trial. Tools that were designed with user-friendly interfaces were found to be more accessible for children and families, lowering barriers to completion. Cook et al. [34] implemented PROMs via a web-based platform with participants most commonly reporting how easy it was to use, that the PROMs could be completed quickly and had the capacity for modification of responses if needed. Participants also reported liking the website aesthetics. Importantly, dyadic reporting approaches, where children and parents could contribute together, were associated with higher acceptability, recognising the value of family involvement in capturing symptom experiences [9]. These strategies, particularly automated reminders, electronic capture and a family-centred approach, demonstrate the potential to strengthen PROM implementation and ensure that symptom experiences are meaningfully integrated into clinical care.

#### 3.3.3. Impact of PROMs on Outcomes

This review revealed some early evidence of potential outcomes and benefits of PROM use in paediatric HSCT care. Whilst PROMs consistently facilitated symptom detection, their impact on clinical outcomes, quality of life, or patient–family experiences was less consistently demonstrated and studied.

##### Patient-Related Outcomes

Symptom-related outcomes

PROMs enabled the early identification of common and burdensome symptoms, including nausea, fatigue, pain, and sleep disruption, with some evidence of improved monitoring when tools were integrated into daily care [17,35]. However, through most studies, PROM data were not linked to measurable reductions in symptom intensity, duration, or distress. Interventional studies, such as those testing exercise or oral care protocols, used PROMs primarily to evaluate the effect of the intervention on symptom intensity, clinical status, and QoL, rather than as a mechanism driving symptom relief [26,37,39].

The use of PROM alerts to remind patients was found to be associated with improved symptom-specific management [17,35]. There was modest evidence of reduction in symptom burden when PROMs were acted upon [40]. Some studies used PROMs to measure outcomes and describe trends across physical and psychological domains, before, during and after interventions [26,37]. The use of PROMs in these studies enabled the monitoring of trends in symptom intensity, occurrence, resolution, and their impact on QoL.

Clinical outcomes

Evidence of PROM-driven improvements in HRQoL or broader clinical outcomes were sparse. Two studies demonstrated associations between symptom burden and HRQoL, but did not establish whether PROM-informed interventions altered these outcomes [29,31]. As such, the causal pathway from PROM reporting informing management strategies that lead to improved outcomes remains underexplored in the paediatric HSCT setting.

##### Symptom Management Outcomes

Satisfaction and acceptability

The successful validation of two PROMs for use in the paediatric oncology context, including inpatient HSCT care, was achieved by two included studies [3,28], which has led to their use in subsequent studies [9,34,35,38] and to the first study to examine the integration of SSPedi into clinical practice [35].

PROM use was generally well received by children and families. Importantly, Tomlinson et al. [9] found that a dyadic approach to symptom screening, where children and parents could contribute together, was associated with higher acceptability, recognising the value of family involvement in capturing symptom experiences. Parents valued the opportunity to contribute to their child’s care, particularly when proxy or dyadic reporting was used. These findings suggest PROMs have the potential to enhance the patient–family care experience, even when their clinical impact remains limited.

Frequency and completion

Cook et al. [34] reported high acceptability of daily electronic symptom reporting, with most participants (93%) considering the frequency “about right”. Ward et al. [31] reported that children from both study cohorts equally reported no difficulty in completing the symptom assessments. In one study, PROM completion rates were found to be higher in participants who had received an autologous transplant when compared to those who had received an allogeneic transplant [17]. Ward et al. [31] reported that children undergoing HSCT completed a higher percentage of administered assessments compared with the non-HSCT cohort.

## 4. Discussion

### 4.1. Introduction to the Discussion

This review identified the PROMs used during inpatient paediatric HSCT and synthesised how these tools contribute to understanding symptom experiences and management. The included studies demonstrate that PROMs can capture a wide range of physical and psychological symptoms experienced during HSCT and are generally feasible and acceptable for children and families. However, their integration into routine inpatient HSCT care remains limited, and the evidence base to date, particularly regarding clinical impact, remains preliminary.

Symptom burden during paediatric HSCT is consistently high and contributes to significant distress, functional disruption and reduced quality of life for children and adolescents through the transplant course [41]. Symptom assessment is the cornerstone of symptom management [42] and PROMs provide an important mechanism for ensuring the voice of the child is represented in symptom assessment [43]. However, their effective integration into clinical practice requires attention to feasibility, workflow, and developmental considerations [44,45]. Although PROMs have demonstrated benefits in adult HSCT and broader paediatric oncology, such as improved communication, enhanced symptom identification, and in some cases reduced symptom burden [7], their use in paediatric HSCT remains in its infancy. Existing studies focus largely on feasibility and tool validation, with limited longitudinal data or evidence of routine clinical integration [3,9,11,17,28,35].

Symptom burden during paediatric HSCT is consistently high and contributes to significant distress, functional disruption, and reduced quality of life for children and adolescents throughout the transplant course. PROMs provide valuable information for the clinical team during symptom assessment and can guide appropriate symptom management strategies for patients. However, effectively implementing PROMs during the acute phase of HSCT requires attention to developmental appropriateness, patient burden, and workflow integration. Although PROMs have demonstrated clear benefits in adult HSCT and in paediatric oncology outpatient and survivorship contexts—such as enhanced communication, earlier symptom identification, and improved patient engagement—routine inpatient use in paediatric HSCT remains uncommon. As a result, most studies to date have focused on feasibility or tool validation rather than demonstrating how PROMs can influence clinical care.

There is a pressing need for a more detailed, continuous understanding of symptom occurrence, timing, and trajectories during acute HSCT admission, as evidence demonstrates that high symptom burden may persist beyond hospitalisation [14]. Existing studies suggest that symptoms peak during predictable periods, yet the scarcity of longitudinal and daily data hinders precise characterisation of these patterns or their clinical implications. Although PROMs appear to strengthen communication and patient engagement, it remains unclear whether their use during acute HSCT can facilitate earlier symptom detection, prompt more responsive management, or improve clinical outcomes. Understanding predictive factors and intervention effects across the transplant continuum is essential to advancing symptom-focused care. Addressing these questions will require studies that not only capture symptom data but also evaluate how PROM-informed interventions affect symptom burden, functioning, and recovery across the transplant continuum.

A persistent challenge is that children experiencing the highest symptom burden are often the least able to complete PROMs [34,38], leading to under-representation of those that are most vulnerable. This pattern mirrors challenges in other high-acuity paediatric settings, where children may be too unwell, fatigued, or overwhelmed to complete assessments, even when symptoms are most severe. These findings underscore the essential role of vigilant nursing advocacy and strong family partnership during HSCT.

A triadic approach to symptom assessment, drawing on perspectives of the child, parent, and nurse, is essential for forming an accurate clinical picture and guiding symptom management [46]. Evidence shows consistent under-reporting of symptoms by caregivers and clinicians, underscoring the need for research to determine how best to capture the child’s true symptom experience, particularly given substantial disagreement between child self-report and care-team assessments [47]. This reinforces the importance of nursing advocacy and family partnership in identifying subtle changes and ensuring timely, evidence-based management. Although age-appropriate PROMs, co-completion methods, and proxy reporting offer strategies to support symptom capture [3,9,28], discrepancies between reporting approaches highlight the complexity of accurately capturing a child’s lived experience through PROMs. Continued refinement and evaluation of these approaches will be essential to improving assessment accuracy and supporting high-quality, symptom-focused care. 

### 4.2. Gaps and Limitations in Current Literature

The current PROM literature in paediatric HSCT is limited by a small number of HSCT-specific studies, with many drawing on broader oncology samples that limit generalisability for the acute transplant setting. Despite the inpatient phase being associated with the highest symptom burden, very few studies capture PROM data during this period, and longitudinal tracking of symptom trajectories across the transplant course remains scarce. Evidence that PROMs inform real-time care or lead to changes in symptom management is minimal, and most studies focus on symptom occurrence and severity rather than distress, meaning, or psychosocial dimensions. Methodological issues such as small sample sizes, inconsistent use of validated tools, and heterogeneity in study designs further limit comparability and generalisability, alongside geographic gaps and limited cultural or linguistic adaptation of PROMs. Overall, the literature lacks integration of PROMs into routine clinical workflows and offers little evidence linking PROM use to actionable management strategies or measurable outcomes. Studies predominantly used PROMs to capture symptom experience, while largely neglecting the evaluation of interventions and their outcomes—two critical components of Symptom Management Theory [22].

### 4.3. Implications for Research and Practice

When mapped to the three components of SMT: symptom experience, management strategies, and outcomes, the current PROM research in paediatric HSCT most comprehensively addresses the symptom experience component, capturing the occurrence and severity of physical and emotional symptoms. In contrast, far fewer studies connected PROMs to real-time management strategies or evaluated their impact on patient outcomes. The evidence base is heavily weighted to symptom identification with limited progression of PROM data to inform targeted management strategies or ultimately influence clinical and QoL outcomes. This mapping reinforces that, although PROMs are beginning to illuminate children’s symptom experiences during HSCT, the field has yet to realise the full SMT pathway that links symptom reporting to responsive symptom-focused care and measurable patient outcomes.

Future research should focus on rigorous, multi-site longitudinal studies to evaluate how PROM-based symptom screening and supportive care interventions affect symptom burden and HRQoL in paediatric HSCT populations [17,18,26,31,34]. PROMs need to be more fully integrated into routine clinical workflows [7], preferably through electronic health record systems with automated alerts, to support timely, personalised symptom management. To ensure PROM data translates into actionable care, clinician training and alignment with evidence-based guidelines are essential. Continued refinement and validation of PROMs, including cultural and linguistic adaptation and approaches that capture distress and symptom meaning, will strengthen their clinical utility [3,18,27]. Addressing barriers such as low completion rates and enhancing electronic data capture methods remain key priorities [28,31,36]. Greater attention to psychosocial screening and supporting multidisciplinary interpretation of PROM results will also be critical [48]. 

### 4.4. Strengths and Limitations

This systematic review draws strength from its adherence to PRISMA reporting guidelines, protocol-guided methodology, comprehensive multi-database search strategy, and transparent screening processes, supported by moderate inter-rater agreement that reinforces the reliability and validity of study selection. Its focus on an under-researched intersection, PROM use during paediatric HSCT inpatient care, adds novelty and clinical relevance, providing insight into a population whose symptom experiences are often underrepresented.

However, several limitations must be acknowledged. The inclusion of mixed-population studies where paediatric HSCT-specific data could not be delineated [3,9,27,28,40], and restriction to English-language peer-reviewed studies limit generalisability. Across included studies, small sample sizes, methodological variability, and a lack of longitudinal data further constrain interpretation [17,32]. Wide heterogeneity in study design, settings, PROM type, and outcome measures also reduces comparability. Together, these limitations highlight the need for more standardised, culturally adaptable PROMs, consistent methodological approaches, and robust longitudinal research to strengthen future evidence and clinical applicability.

## 5. Conclusions

Although PROMs are well established in adult oncology/HSCT and have gained traction over the last decade in paediatric oncology, outpatient and survivorship contexts, their systematic integration into routine inpatient paediatric HSCT care remains limited. While current evidence suggests they can effectively capture symptom experience, their routine use, guidance of symptom management, or influence of patient outcomes in paediatric HSCT is still limited and largely unrealised. Current evidence does not demonstrate consistent or causal improvements in clinical outcomes, and research to date remains exploratory, with limited HSCT-specific data and minimal integration of PROMs into real-time inpatient care. Future work should prioritise longitudinal, implementation-focused studies that evaluate not only symptom detection but also whether PROM-driven interventions can meaningfully improve the clinical experiences and outcomes of children undergoing HSCT.

## Data Availability

The original contributions presented in this study are included in the article/Supplementary Materials. Further inquiries can be directed to the corresponding authors.

## Supplementary Materials

The following supporting information can be downloaded at: https://www.mdpi.com/article/10.3390/children13040491/s1, Table S1: Characteristics of PROMs used in included studies.

## Abbreviations

The following abbreviations are used in this manuscript:

CHIMES	Children’s International Mucositis Evaluation Scale
ERM	Electronic Medical Record
FACES	Wong–Baker FACES Pain Rating Scale
FPS-R	Faces Pain Scale–Revised
GQoL	Global Quality of Life visual categorical scale
HRQoL	Health Related Quality of Life
HSCT	Haematopoietic Stem Cell Transplant
MDASI-Adol	MD Anderson Symptom Inventory–adolescent
MSAS	Memorial Symptom Assessment Scale
OHIP-14	Short form of the Oral Health Impact Profile
Peds QL	Peds Quality of Life
Peds QL CM	Peds Quality of Life Cancer Module
PeNAT	Pediatric Nausea Assessment Tool
PQ-MSAS-	The Pediatric Quality of Life and evaluation of Symptoms study
PRISMA	Preferred Reporting Items for Systematic Reviews and Meta-Analyses
PROMs	Patient-Reported Outcome Measures
PROMIS	Patient Reported Outcomes Measurement Information System
PROMS	Patient-Reported Oral Mucositis Symptom
QoL	Quality of Life
QuADS	Quality Assessment for Diverse Studies
SMT	Symptom Management Theory
SSPedi	Symptom Screening in Pediatrics Tool

## Appendix A

**Table A1:** Search strategy.

Search Strategy: The use of patient reported outcome measures to inform clinical care in paediatric haematopoietic stem cell transplant			
Database	**Medline via EBSCO Host**		
Date	**2 October 2024**		
MH	S1	(MH “Pediatrics+”)	19,244
Title or Abstract	S2	TI (P*ediatric OR child* OR children OR infant OR adolescent OR youth OR ‘young person’ OR teenager OR ‘young adult’) OR AB (P*ediatric OR child* OR children OR infant OR adolescent OR youth OR ‘young person’ OR teenager OR ‘young adult’)	13,229,989
	S3	(MH “Symptom Assessment”)	6577
	S4	(MH “Patient Outcome Assessment+”)	25,142
	S5	(MH “Nursing Assessment+”)	2674
	S6	(MH “Patient Reported Outcome Measures+”)	16,363
	S7	TI (“Patient assessment*” OR “Outcome assessment” OR “Assessment tool*” OR “clinical assessment tool*” OR “Assessment model*” OR “Symptom assessment*” OR “Symptom assessment scale” OR “Symptom screening” OR “Nursing assessment*” OR “Nursing evaluation” OR “Toxicity assessment*” OR “Toxicity screening” OR “Patient outcome assessment*” OR “Patient-reported experience*” OR “Patient-reported outcome*” OR “Patient-reported outcome measure*” OR “Health screening” OR “Patient reported experience*” OR “Patient reported outcome*” OR “Patient reported outcome measure*” OR “Symptom checklist*”) OR AB (“Patient assessment*” OR “Outcome assessment” OR “Assessment tool*” OR “clinical assessment tool*” OR “Assessment model*” OR “Symptom assessment*” OR “Symptom assessment scale” OR “Symptom screening” OR “Nursing assessment” OR “Nursing evaluation*” OR “Toxicity assessment” OR “Toxicity screening” OR “Patient outcome assessment*” OR “Patient-reported experience*” OR “Patient-reported outcome*” OR “Patient-reported outcome measure*” OR “Health screening” OR “Patient reported experience*” OR “Patient reported outcome*” OR “Patient reported outcome measure*”OR “Symptom checklist*”)	90,445
	S8	(MH “Bone Marrow Transplantation”)	4838
	S9	(MH “Mesenchymal Stem Cell Transplantation”)	10,514
	S10	(MH “Hematopoietic Stem Cell Transplantation+”)	30,290
	S11	(MH “Stem Cell Transplantation+”)	50,866
	S12	(MH “Peripheral Blood Stem Cell Transplantation”)	1098
	S13	(MH “Cord Blood Stem Cell Transplantation”)	1667
	S14	TI (“Bone Marrow*” OR “Stem Cell” OR “Bone marrow transplant*” OR BMT OR “H*ematopoietic Stem Cell Transplant*” OR HSCT or “Stem Cell Transplant*” OR SCT OR “Cord Blood Stem Cell Transplant*” OR “Peripheral Blood Stem Cell Transplant*” OR “Allogenic bone marrow transplant*” OR “Autologous bone marrow transplant*” OR “mesenchymal stem cell transplant*” OR “MSC transplant*” OR “multipotent mesenchymal stromal cell transplant*”) OR AB (“Bone marrow transplant*” OR BMT OR “H*ematopoietic Stem Cell Transplant*” OR HSCT or “Stem Cell Transplant*” OR SCT OR “Cord Blood Stem Cell Transplant*” OR “Peripheral Blood Stem Cell Transplant*” OR “Allogenic bone marrow transplant*” OR “Autologous bone marrow transplant*” OR “mesenchymal stem cell transplant*” OR “MSC transplant*” OR “ multipotent mesenchymal stromal cell transplant*”)	95,248
Strategy	e.g., 1 AND #2 AND #3		
S15	S1 OR S2		13,231,253
S16	S3 OR S4 OR S5 OR S6 OR S7		110,268
S17	S8 OR S9 OR S10 OR S11 OR S12 OR S13 OR S14		118,031
S18	S15 AND S16 AND S17		755
Database	**CINAHL** via EBSCO Host		
Date	**2 October 2024**		
MH	S1	(MH “Pediatrics+”)	11,602
Title or Abstract	S2	TI (P*ediatric OR child* OR children OR infant OR adolescent OR youth OR ‘young person’ OR teenager OR ‘young adult’) OR AB (P*ediatric OR child* OR children OR infant OR adolescent OR youth OR ‘young person’ OR teenager OR ‘young adult’)	3,642,086
	S3	(MH “Patient Assessment+”)	29,368
	S4	(MH “Outcome Assessment”)	30,684
	S5	(MH “Nursing Assessment”)	3775
	S6	(MH “Patient-Reported Outcomes+”)	8333
	S7	(MH “Clinical Assessment Tools+”)	150,342
	S8	TI (“Patient assessment*” OR “Outcome assessment” OR “Assessment tool*” OR “clinical assessment tool*” OR “Assessment model*” OR “Symptom assessment*” OR “Symptom assessment scale” OR “Symptom screening” OR “Nursing assessment*” OR “Nursing evaluation” OR “Toxicity assessment*” OR “Toxicity screening” OR “Patient outcome assessment*” OR “Patient-reported experience*” OR “Patient-reported outcome*” OR “Patient-reported outcome measure*” OR “Health screening” OR “Patient reported experience*” OR “Patient reported outcome*” OR “Patient reported outcome measure*” OR “Symptom checklist*”) OR AB (“Patient assessment*” OR “Outcome assessment” OR “Assessment tool*” OR “clinical assessment tool*” OR “Assessment model*” OR “Symptom assessment*” OR “Symptom assessment scale” OR “Symptom screening” OR “Nursing assessment” OR “Nursing evaluation*” OR “Toxicity assessment” OR “Toxicity screening” OR “Patient outcome assessment*” OR “Patient-reported experience*” OR “Patient-reported outcome*” OR “Patient-reported outcome measure*” OR “Health screening” OR “Patient reported experience*” OR “Patient reported outcome*” OR “Patient reported outcome measure*”OR “Symptom checklist*”)	39,060
	S9	(MH “Bone Marrow Transplantation+”)	1412
	S10	(MH “Bone Marrow Transplantation, Allogeneic”)	152
	S11	(MH “Bone Marrow Transplantation, Autologous”)	123
	S12	(MH “Hematopoietic Stem Cell Transplantation”)	6327
	S13	TI (“Bone Marrow*” OR “Stem Cell” OR “Bone marrow transplant*” OR BMT OR “H*ematopoietic Stem Cell Transplant*” OR HSCT or “Stem Cell Transplant*” OR SCT OR “Cord Blood Stem Cell Transplant*” OR “Peripheral Blood Stem Cell Transplant*” OR “Allogenic bone marrow transplant*” OR “Autologous bone marrow transplant*” OR “mesenchymal stem cell transplant*” OR “MSC transplant*” OR “multipotent mesenchymal stromal cell transplant*”) OR AB (“Bone marrow transplant*” OR BMT OR “H*ematopoietic Stem Cell Transplant*” OR HSCT or “Stem Cell Transplant*” OR SCT OR “Cord Blood Stem Cell Transplant*” OR “Peripheral Blood Stem Cell Transplant*” OR “Allogenic bone marrow transplant*” OR “Autologous bone marrow transplant*” OR “mesenchymal stem cell transplant*” OR “MSC transplant*” OR “ multipotent mesenchymal stromal cell transplant*”)	16,344
Strategy	e.g., 1 AND #2 AND #3		
S14	S1 OR S2		3,643,523
S15	S3 OR S4 OR S5 OR S6 OR S7 OR S8		234,629
S16	S9 OR S10 OR S11 OR S12 OR S13		18,928
S17	S14 AND S15 AND S16		1006
Database	**APA PsycINFO via EBSCO host**		
Date	**2 October 2024**		
MH	S1	MM “Pediatrics”	13,871
Title or Abstract	S2	TI (P*ediatric OR child* OR children OR infant OR adolescent OR youth OR ‘young person’ OR teenager OR ‘young adult’) OR AB (P*ediatric OR child* OR children OR infant OR adolescent OR youth OR ‘young person’ OR teenager OR ‘young adult’)	1,969,221
	S3	MM “Symptom Checklists”	235
	S4	MM “Patient Reported Outcome Measures”	701
	S5	TI (“Patient assessment*” OR “Outcome assessment” OR “Assessment tool*” OR “clinical assessment tool*” OR “Assessment model*” OR “Symptom assessment*” OR “Symptom assessment scale” OR “Symptom screening” OR “Nursing assessment*” OR “Nursing evaluation” OR “Toxicity assessment*” OR “Toxicity screening” OR “Patient outcome assessment*” OR “Patient-reported experience*” OR “Patient-reported outcome*” OR “Patient-reported outcome measure*” OR “Health screening” OR “Patient reported experience*” OR “Patient reported outcome*” OR “Patient reported outcome measure*” OR “Symptom checklist*”) OR AB (“Patient assessment*” OR “Outcome assessment” OR “Assessment tool*” OR “clinical assessment tool*” OR “Assessment model*” OR “Symptom assessment*” OR “Symptom assessment scale” OR “Symptom screening” OR “Nursing assessment” OR “Nursing evaluation*” OR “Toxicity assessment” OR “Toxicity screening” OR “Patient outcome assessment*” OR “Patient-reported experience*” OR “Patient-reported outcome*” OR “Patient-reported outcome measure*” OR “Health screening” OR “Patient reported experience*” OR “Patient reported outcome*” OR “Patient reported outcome measure*”OR “Symptom checklist*”)	16,907
	S6	MM “Bone Marrow”	224
	S7	MM “Stem Cells”	2281
	S8	TI (“Bone Marrow*” OR “Stem Cell” OR “Bone marrow transplant*” OR BMT OR “H*ematopoietic Stem Cell Transplant*” OR HSCT or “Stem Cell Transplant*” OR SCT OR “Cord Blood Stem Cell Transplant*” OR “Peripheral Blood Stem Cell Transplant*” OR “Allogenic bone marrow transplant*” OR “Autologous bone marrow transplant*” OR “mesenchymal stem cell transplant*” OR “MSC transplant*” OR “multipotent mesenchymal stromal cell transplant*”) OR AB (“Bone marrow transplant*” OR BMT OR “H*ematopoietic Stem Cell Transplant*” OR HSCT or “Stem Cell Transplant*” OR SCT OR “Cord Blood Stem Cell Transplant*” OR “Peripheral Blood Stem Cell Transplant*” OR “Allogenic bone marrow transplant*” OR “Autologous bone marrow transplant*” OR “mesenchymal stem cell transplant*” OR “MSC transplant*” OR “ multipotent mesenchymal stromal cell transplant*”)	2467
Strategy	e.g., 1 AND #2 AND #3		
S9	S1 OR S2		1,969,221
S10	S3 OR S4 OR S5		17,147
S11	S6 OR S7 OR S8		3916
S12	S9 AND S10 AND S11		49
Database	**Cochrane Library**		
Date	2 October 2024		
MH	S2	MH “Pediatrics”	163
Title or Abstract	S1	TI (P?ediatric OR child* OR children OR infant OR adolescent OR youth OR ‘young person’ OR teenager OR ‘young adult’) OR AB (P?ediatric OR child* OR children OR infant OR adolescent OR youth OR ‘young person’ OR teenager OR ‘young adult’)	3444
	S3	(MH “Symptom Assessment”)	20
	S4	(MH “Patient Outcome Assessment+”)	7
	S5	(MH “Outcome Assessment”)	1259
	S6	(MH “Nursing Assessment”)	0
	S7	(MH “Patient Reported Outcome Measures+”)	41
	S8	(MH “Patient reported outcome”)	148
	S9	TI ((Patient NEXT assessment*) OR (Outcome NEXT assessment) OR (Assessment NEXT tool*) OR (clinical NEXT assessment NEXT tool*) OR (Assessment NEXT model*) OR (Symptom NEXT assessment*) OR (Symptom NEXT assessment NEXT scale) OR (Symptom NEXT screening) OR (Nursing NEXT assessment*) OR (Nursing NEXT evaluation) OR (Toxicity NEXT assessment*) OR (Toxicity NEXT screening) OR (Patient NEXT outcome NEXT assessment*) OR (Patient-reported NEXT experience*) OR (Patient-reported NEXT outcome*) OR (Patient-reported NEXT outcome NEXT measure*) OR (Health NEXT screening) OR (Patient NEXT reported NEXT experience*) OR (Patient NEXT reported NEXT outcome*) OR (Patient NEXT reported NEXT outcome NEXT measure*) OR (Symptom NEXT checklist*)) OR AB ((Patient NEXT assessment*) OR (Outcome NEXT assessment) OR (Assessment NEXT tool*) OR (clinical NEXT assessment NEXT tool*) OR (Assessment NEXT model*) OR (Symptom NEXT assessment*) OR (Symptom NEXT assessment NEXT scale) OR (Symptom NEXT screening) OR (Nursing NEXT assessment*) OR (Nursing NEXT evaluation) OR (Toxicity NEXT assessment*) OR (Toxicity NEXT screening) OR (Patient NEXT outcome NEXT assessment*) OR (Patient-reported NEXT experience*) OR (Patient-reported NEXT outcome*) OR (Patient-reported NEXT outcome NEXT measure*) OR (Health NEXT screening) OR (Patient NEXT reported NEXT experience*) OR (Patient NEXT reported NEXT outcome*) OR (Patient NEXT reported NEXT outcome NEXT measure*) OR (Symptom NEXT checklist*)) OR AB ((Patient NEXT assessment*) OR (Outcome NEXT assessment) OR (Assessment NEXT tool*) OR (clinical NEXT assessment NEXT tool*) OR (Assessment NEXT model*) OR (Symptom NEXT assessment*) OR (Symptom NEXT assessment NEXT scale) OR (Symptom NEXT screening) OR (Nursing NEXT assessment*) OR (Nursing NEXT evaluation) OR (Toxicity NEXT assessment*) OR (Toxicity NEXT screening) OR (Patient NEXT outcome NEXT assessment*) OR (Patient-reported NEXT experience*) OR (Patient-reported NEXT outcome*) OR (Patient-reported NEXT outcome NEXT measure*) OR (Health NEXT screening) OR (Patient NEXT reported NEXT experience*) OR (Patient NEXT reported NEXT outcome*) OR (Patient NEXT reported NEXT outcome NEXT measure*) OR (Symptom NEXT checklist*)) OR AB ((Patient NEXT assessment*) OR (Outcome NEXT assessment) OR (Assessment NEXT tool*) OR (clinical NEXT assessment NEXT tool*) OR (Assessment NEXT model*) OR (Symptom NEXT assessment*) OR (Symptom NEXT assessment NEXT scale) OR (Symptom NEXT screening) OR (Nursing NEXT assessment*) OR (Nursing NEXT evaluation) OR (Toxicity NEXT assessment*) OR (Toxicity NEXT screening) OR (Patient NEXT outcome NEXT assessment*) OR (Patient-reported NEXT experience*) OR (Patient-reported NEXT outcome*) OR (Patient-reported NEXT outcome NEXT measure*) OR (Health NEXT screening) OR (Patient NEXT reported NEXT experience*) OR (Patient NEXT reported NEXT outcome*) OR (Patient NEXT reported NEXT outcome NEXT measure*) OR (Symptom NEXT checklist*))	1822
	S10	(MH Bone Marrow Transplantation)	43
	S11	(MH Mesenchymal Stem Cell Transplantation)	13
	S12	(MH Hematopoietic Stem Cell Transplantation)	79
	S13	(MH Stem Cell Transplantation)	144
	S14	(MH Peripheral Blood Stem Cell Transplantation)	21
	S15	(MH Cord Blood Stem Cell Transplantation)	2
	S16	TI ((Bone NEXT Marrow*) OR (Stem NEXT Cell) OR (Bone NEXT marrow NEXT transplant*) OR BMT OR (H?ematopoietic NEXT Stem NEXT Cell NEXT Transplant*) OR HSCT or (Stem NEXT Cell NEXT Transplant*) OR SCT OR (Cord NEXT Blood NEXT Stem NEXT Cell NEXT Transplant*) OR (Peripheral NEXT Blood NEXT Stem NEXT Cell NEXT Transplant*) OR (Allogenic NEXT bone NEXT marrow NEXT transplant*) OR (Autologous NEXT bone NEXT marrow NEXT transplant*) OR (mesenchymal NEXT stem NEXT cell NEXT transplant*) OR (MSC NEXT transplant*) OR (multipotent NEXT mesenchymal NEXT stromal NEXT cell NEXT transplant*)) OR AB ((Bone NEXT Marrow*) OR (Stem NEXT Cell) OR (Bone NEXT marrow NEXT transplant*) OR BMT OR (H?ematopoietic NEXT Stem NEXT Cell NEXT Transplant*) OR HSCT or (Stem NEXT Cell NEXT Transplant*) OR SCT OR (Cord NEXT Blood NEXT Stem NEXT Cell NEXT Transplant*) OR (Peripheral NEXT Blood NEXT Stem NEXT Cell NEXT Transplant*) OR (Allogenic NEXT bone NEXT marrow NEXT transplant*) OR (Autologous NEXT bone NEXT marrow NEXT transplant*) OR (mesenchymal NEXT stem NEXT cell NEXT transplant*) OR (MSC NEXT transplant*) OR (multipotent NEXT mesenchymal NEXT stromal NEXT cell NEXT transplant*))	425
Strategy	e.g., 1 AND #2 AND #3		
S17	#1 OR #2		3603
S18	#3 OR #4 OR #5 OR #6 OR #7 OR #8 OR #9		3143
S19	#10 OR #11 OR #12 OR #13 OR #14 OR #15 OR #16		570
S20	#17 AND #18 AND #19		17
Database	Embase		
Date	2 October 2024		
MH	#3	‘pediatrics’/de AND [embase]/lim AND [2014–2024]/py	42,965
Title or Abstract	#1	(ti AND (p?ediatric OR child* OR children OR infant OR adolescent OR youth OR ‘young person’ OR teenager OR ‘young adult’) OR ab) AND (p?ediatric OR child* OR children OR infant OR adolescent OR youth OR ‘young person’ OR teenager OR ‘young adult’) AND [2014–2024]/py	36,494
	#5	‘symptom assessment’/de AND [2014–2024]/py	11,967
	#2	‘symptom assessment scale’ AND [2014–2024]/py	1543
	#4	‘patient assessment’/de AND [2014–2024]/py	15,229
	#6	‘symptom checklist’/de AND [2014–2024]/py	15
	#7	‘outcome assessment’ AND [2014–2024]/py	683,985
	#8	‘nursing assessment’/de AND [2014–2024]/py	2004
	#9	‘patient reported outcome measure’/de AND [2014–2024]/py	107
	#10	‘patient-reported outcome’/de AND [2014–2024]/py	65,477
	#11	‘clinical assessment tool’/de AND [2014–2024]/py	15,285
	#12	(ti AND (‘patient assessment*’ OR ‘outcome assessment’ OR ‘assessment tool*’ OR ‘clinical assessment tool*’ OR ‘assessment model*’ OR ‘symptom assessment*’ OR ‘symptom assessment scale’ OR ‘symptom screening’ OR ‘nursing assessment*’ OR ‘nursing evaluation’ OR ‘toxicity assessment*’ OR ‘toxicity screening’ OR ‘patient outcome assessment*’ OR ‘patient-reported experience*’ OR ‘patient-reported outcome*’ OR ‘patient-reported outcome measure*’ OR ‘health screening’ OR ‘patient reported experience*’ OR ‘patient reported outcome*’ OR ‘patient reported outcome measure*’ OR ‘symptom checklist*’) OR ab) AND (‘patient assessment*’ OR ‘outcome assessment’ OR ‘assessment tool*’ OR ‘clinical assessment tool*’ OR ‘assessment model*’ OR ‘symptom assessment*’ OR ‘symptom assessment scale’ OR ‘symptom screening’ OR ‘nursing assessment’ OR ‘nursing evaluation*’ OR ‘toxicity assessment’ OR ‘toxicity screening’ OR ‘patient outcome assessment*’ OR ‘patient-reported experience*’ OR ‘patient-reported outcome*’ OR ‘patient-reported outcome measure*’ OR ‘health screening’ OR ‘patient reported experience*’ OR ‘patient reported outcome*’ OR ‘patient reported outcome measure*’ OR ‘symptom checklist*’) AND [2014–2024]/py	12,490
	#13	‘bone marrow transplantation’/de AND [2014–2024]/py	16,591
	#14	‘allogenic bone marrow transplantation’/de AND [2014–2024]/py	2202
	#15	‘autologous bone marrow transplantation’/de AND [2014–2024]/py	1031
	#16	‘mesenchymal stem cell transplantation’/de AND [2014–2024]/py	10,237
	#17	‘hematopoietic stem cell transplantation’/de AND [2014–2024]/py	31,106
	#18	‘stem cell transplantation’/de AND [2014–2024]/py	23,739
	#19	‘peripheral blood stem cell transplantation’/de AND [2014–2024]/py	1647
	#20	‘cord blood stem cell transplantation’/de AND [2014–2024]/py	3876
	#21	(ti AND (‘bone marrow*’ OR ‘stem cell’ OR ‘bone marrow transplant*’ OR bmt OR ‘h?ematopoietic stem cell transplant*’ OR hsct OR ‘stem cell transplant*’ OR sct OR ‘cord blood stem cell transplant*’ OR ‘peripheral blood stem cell transplant*’ OR ‘allogenic bone marrow transplant*’ OR ‘autologous bone marrow transplant*’ OR ‘mesenchymal stem cell transplant*’ OR ‘msc transplant*’ OR ‘multipotent mesenchymal stromal cell transplant*’) OR ab) AND (‘bone marrow transplant*’ OR bmt OR ‘h?ematopoietic stem cell transplant*’ OR hsct OR ‘stem cell transplant*’ OR sct OR ‘cord blood stem cell transplant*’ OR ‘peripheral blood stem cell transplant*’ OR ‘allogenic bone marrow transplant*’ OR ‘autologous bone marrow transplant*’ OR ‘mesenchymal stem cell transplant*’ OR ‘msc transplant*’ OR ‘multipotent mesenchymal stromal cell transplant*’)	3898
Strategy	e.g., 1 AND #2 AND #3		
#22	#3 OR #1		78,881
#23	#5 OR #2 OR #4 OR #6 OR #7 OR #8 OR #9 OR #10 OR #11 OR #12		765,341
#24	#13 OR #14 OR #15 OR #16 OR #17 OR #18 OR #19 OR #20 OR #21		86,633
#25	#22 AND #23 AND #24		144

## Appendix B

**Table A2:** Characteristics of included studies.

Author, Year, and Location	Setting	Study Design and Aim	Study Population Characteristics	PROM Used to Assess Symptoms
Bezinelli, Eduardo [26] Brazil	Single-centre; private, tertiary hospital	Study design Prospective cohort study Aim To describe Quality of Life (QoL) related to mucositis among patients undergoing Hematopoietic Stem Cell Transplant (HSCT), and mucositis prevention and treatment with specialised oral care and low-level laser therapy.	Total number of participants *n* (%) 69 (100) 9 (13.04) aged up to 17 years HSCT recipients *n* (%) 69 (100) total 9 (13.04) aged up to 17 years Type of cancer *n* (%) Multiple myeloma: 20 (28.99) * AML: 12 (17.39) ** ALL: 7 (10.14) *** NHL: 7 (10.14) **** HL: 5 (7.25) ***** MDS: 4 (5.8) Other: 14 (20.2) Demographics Age in years, *n* (%) Up to 17 years: 9 (13.04) 18–35 years: 20 (28.99) 36–59 years: 16 (23.19) Over 60: 24 (34.78) Sex—Male *n* (%) 46 (66.67)	Patient-Reported Oral Mucositis Symptom (PROMS) scale Short form of the Oral Health Impact Profile (OHIP-14)
Cook, Vettese [34] Canada	Single-centre; paediatric cancer and HSCT	Study design Randomized controlled trial. Aim To determine the feasibility of a future randomized controlled trial of daily symptom screening for 5 days among paediatric inpatients and outpatients with cancer or HSCT recipients.	Total number of participants 30 HSCT recipients *n* (%) 10 (33.3) Type of cancer *n* (%) Leukaemia/Lymphoma: 17 (56.7) Solid tumor: 8 (26.7) Brain tumor: 0 (0.0) Other (non-malignant): 5 (16.7%) Relapse: 9 (30.0) Demographics Age in years *n* (%) 8–10 years: 10 (33.3) 11–14 years: 10 (33.3) 15–18 years: 10 (33.3) Sex—Male *n* (%) 13 (43.3)	Symptom Screening in Pediatrics Tool (SSPedi)
Dupuis, Johnston [3] Canada and United States of America	Nine sites, inpatient, and outpatient setting	Study design Prospective cohort study Aim To evaluate the reliability and validity of self-report SSPedi from the perspective of children with cancer and pediatric HSCT recipients.	Total number of participants Total: 502 ‘More symptomatic’ group which included HSCT recipients: 302. HSCT recipients *n* (%) Total: 32 (6.4) ‘More symptomatic’ group: 32 (10.6) Type of cancer *n* (%) Leukaemia/Lymphoma: 354 (70.5) Solid tumor: 122 (24.3) Brain tumor: 18 (3.6) Other: 8 (1.6) Demographics Age in years (median, IQR) 8–10 years: 150 (29.9) 11–14 years: 202 (40.2) 15–18 years: 150 (29.9) Sex—Male *n* (%) 308 (61.4)	SSPedi Global Quality of Life (QoL) visual categorical scale Children’s International Mucositis Evaluation Scale (ChIMES) Faces Pain Scale–Revised (FPS-R) Paediatric Nausea Assessment Tool (PeNAT) 5-point global symptom change scale
Dupuis, Johnston [35] Canada	Eight tertiary care centers	Study design Randomized controlled trial. Aim To determine if daily symptom screening and provision of symptom reports to the health care team was associated with lower total symptom burden.	Total number of participants 345 total Symptom Screening group: 176 Usual Care group: 169 HSCT recipients *n* (%) Symptom screening group 19 (10.8) Usual care group 14 (8.3) Type of cancer Symptom screening group Leukemia: 80 (45.5) Lymphoma: 36 (20.5) Solid tumor: 49 (27.8) Brain tumor: 11 (6.2) Demographics Age in years, median (IQR)-Symptom screening group 8–10 years: 45 (25.6) 11–14 years: 69 (39.2) 15–18 years: 62 (35.2) Age in years, median (IQR)- Usual care group 8–10 years: 42 (24.9) 11–14 years: 70 (41.4) 15–18 years: 57 (33.7) Sex—Male *n* (%) Symptom screening, 100 (56.8) Usual care, 95 (56.2)	SSPedi Face Pain Scale–Revised (FPR-R) PedsQL 3.0 Acute Cancer Module
Ford, Vaughn [32] United States of America	Paediatric HSCT	Study design Pilot study Aim To develop a biopsychosocial assessment clinical analytic tool to examine symptom relationships for children undergoing BMT to find actionable relationships for intervention to improve clinical outcomes including mood.	Total number of participants 4 HSCT recipients *n* (%) 4 (100%) Type of cancer Not reported Demographics Age in years, *n* (%) 11–14 years: 4 (100) Sex—Male *n* (%) 2 (50)	mHealth application
Hetzer, Meryk [17] Austria	Single-centre; paediatric HSCT	Study design Prospective, longitudinal cohort study Aim To implement and evaluate feasibility of capturing daily patient-reported outcomes (PROs) in the acute phase of SCT to measure physical and psychosocial symptom burden.	Total number of participants 20 total Auto-SCT Group: *n* = 9 Allo-SCT Group: *n* = 11 HSCT recipients *n* (%) Auto-SCT Group: 9 (45) Allo-SCT Group: 11 (55) Type of cancer *n* (%) Auto-SCT Group Malignant: 9 (100) Non-malignant: 0 (0.0) Allo-SCT Group Malignant: 5 (45.5) Non-malignant: 6 (54.5) Demographics Age in years, median (IQR) Auto-SCT Group: 6.1 (2.2–15.4) Allo-SCT Group: 10.3 (4.9–16.1) Sex—Male *n* (%) Auto-SCT Group: 4 (44.4) Allo-SCT Group: 4 (36.4)	ePROtect
Johnston, Gerbing [36] Canada and United States of America	Paediatric oncology & HSCT	Study design Prospective cohort study Aim To determine the impact of a patient-reported outcome (PRO) coordinator on HRQL questionnaire completion rates during a pediatric acute myeloid leukemia trial.	Total number of participants 231 (67 of these were removed) HSCT recipients *n* (%) Not reported Type of cancer Not reported Demographics Not reported	PedsQL 4.0 Generic Core Scales PedsQL 3.0 Cancer Module PedsQL Multidimensional Fatigue Scale
Rodgers, Wills-Bagnato [29] United States of America	Single-centre; paediatric HSCT	Study design Prospective cohort study—repeat measures design Aim The primary aim of this study is to describe the HRQoL changes over time of children and adolescents by month during the first 6 months post-allogeneic HSCT. A secondary aim is to estimate the association between mean HRQoL and demographic information (age, sex, ethnicity), diagnosis, transplant factors (stem cell source and match), and individual symptom presence reported at 1 month post-allogeneic HSCT.	Total number of participants 23 HSCT recipients *n* (%) 23 (100) Type of cancer *n* (%) Malignant: 16 (69.6) Non-malignant: 7 (30.4) Demographics Age in years, *n* (%) 7–12 years: 14 (60.9) 13–18 years: 9 (39.1) Sex—Male *n* (%) 15 (65.2)	Memorial Symptom Assessment Scale (MSAS) Peds Quality of Life Cancer Module (PedsQL CM™)
Rodgers, Highberger [18] United States of America	Three paediatric teaching hospitals; paediatric HSCT	Study design Prospective study—repeat measures design Aim The primary aim is to describe symptom incidence, severity, and distress trajectories among adolescents from pre-HSCT through 90 days post HSCT. A secondary aim is to examine the relationship between symptom trajectories and demographic and treatment factors.	Total number of participants 58 HSCT recipients *n* (%) 58 (100) Type of cancer *n* (%) Malignant: 40 (69) Non-malignant: 18 (31) Demographics Age in years, *n* (%) 10–12 years: 18 (31) 13–15 years: 21 (36) 16–19 years: 19 (33) Sex—Male *n* (%) 30 (52)	Memorial Symptom Assessment Scale 10–18 (MSAS 10–18)
Sheikh, Miller [27] United States of America	Children’s cancer hospital, HSCT inpatient unit	Study design Prospective cohort study Aim To determine the utility of the MDASI-Adol in the AYA HSCT population to describe the symptom burden and the resulting symptom interferences experienced by patients in the peri-transplant period as well as to describe the interventions/consultations performed due to the data gathered from the MDASI-Adol.	Total number of participants 21 HSCT recipients *n* (%) 21 (100) Type of cancer *n* (%) ALL: 7 (33) AML: 6 (28) Mixed lineage leukaemia: 1 (5) HL: 3 (14) MDS: 1 (5) Medulloblastoma: 1 (5) Non-malignant conditions: 2 (10) Demographics Age in years (mean, range) 20 (13–25) Sex—Male *n* (%) 12 (57)	MD Anderson Symptom Inventory–adolescent (MDASI- Adol)
Smith, Farhat [37] United States of America	Single-centre; paediatric HSCT	Study design Prospective randomized controlled trial Aim To evaluate the effects of a supervised aerobic and resistance exercise program on functional ability, mobility, strength, and QoL during and following pediatric HSCT	Total number of participants 40 total Control group: *n* = 20 Intervention group: *n* = 20 HSCT recipients *n* (%) 40 (100) Type of cancer *n* (%) Total malignancy: 24 (60.0) Control group: 13 (65.0) Intervention group: 11 (55.5) Demographics Age (median, IQR) 13.5 (9–15) overall 13.5 (10–15.5) Control group 13.5 (7.5–15) Intervention group Sex—Male *n* (%) 20 (50) overall 10 (50) Control group 10 (50) Intervention group	Patient-Reported Outcomes Measurement Information System (PROMIS)
Tomlinson, Tardif-Theriault [9] Canada	Single-centre	Study design Randomized controlled trial. Aim The primary objective is to compare co-SSPedi scores vs. proxy-report (proxy-SSPedi) and self-report (SSPedi/mini-SSPedi) scores for pediatric patients receiving cancer treatments. The secondary objectives are: (1) to describe co-SSPedi, proxy SSPedi, and self-report SSPedi variability (represented by score standard deviations); (2) among those randomized to proxy-report and self-report first, to compare within-person severely bothersome scores; and (3) to describe preferences for SSPedi type.	Total number of participants 420 total Co-SSPedi group: 213 Proxy-or self-reported SSPedi group: 207 HSCT recipients *n* (%) Co-SSpedi completion: 29 (13.6%) Proxy-or self-reported SSPedi: 27 (13.0) Type of cancer *n* (%) Co-SSPedi group Leukemia: 93 (43.7) Lymphoma: 35 (16.4) Solid tumor: 51 (23.9) Brain tumor: 23 (10.3) Not cancer: 11 (5.2) Proxy- or self-reported SSpedi group Leukemia:87 (42.0) Lymphoma: 25 (12.1) Solid tumor: 55 (26.6) Brain tumor: 24 (10.6) Not cancer: 16 (7.7) Demographics Age (median, IQR) Co-SSPedi group 4–7 years: 64 (30.0) 8–10 years: 40 (18.8) 11–14 years: 64 (30.0) 15–18 years: 45 (21.1) Proxy- or self-reported SSpedi group 4–7 years: 63 (30.4) 8–10 years: 38 (18.4) 11–14 years: 58 (28.0) 15–18 years: 48 (23.2) Sex—Male *n* (%) Co-SSPedi group: 121 (56.8) Proxy- or self-reported SSpedi group: 125 (60.4)	Co-SSPedi Proxy SSPedi/Self-reported SSPedi
Tomlinson, Dupuis [28] Canada	Three paediatric sites	Study design Prospective cohort study Aim To determine whether co-SSPedi is reliable and valid in paediatric patients receiving cancer treatments or hematopoietic cell transplant.	Total number of participants 501 (301 in the ‘more symptomatic’ which included HSCT patients) HSCT recipients *n* (%) 39 (7.8) Type of cancer Leukemia: 260 (51.9) Lymphoma: 56 (11.2) Solid tumor: 113 (22.6) Brain tumor: 48 (9.6) Other: 24 (4.8) Demographics Age in years, *n* (%) 4–7 years: 165 (32.9) 8–10 years: 96 (19.2) 11–14 years: 140 (27.9) 15–18 years: 100 (20.0) Sex—Male *n* (%) 283 (56.5)	Co-Symptom Screening in Pediatrics Tool (Co-SSPedi)
Vettese, Cook [38] Canada and United States of America	Nine sites	Study design Longitudinal, single-armed feasibility study Aim To determine the feasibility of a future randomized trial of longitudinal symptom screening by describing the proportion of children who completed symptom screening on at least 60% of on-study days during a five-day longitudinal study and to refine SPARK to improve longitudinal utilization from the perspective of children receiving cancer treatments or undergoing HSCT.	Total number of participants 30 HSCT recipients *n* (%) 5 (16.7) Type of cancer Leukemia/lymphoma: 16 (53.3) Solid tumor:11 (36.7) Brain tumor: 0 (0.0) Other: 3 (10.0) Demographics Age in years, *n* (%) 8–10 years: 8 (26.7) 11–14 years: 11 (36.7) 15–18 years: 11 (36.7) Sex—Male *n* (%) 18 (60.0)	SSPedi
Ward, Balian [31] United States of America	Five paediatric cancer centres	Study design Prospective cohort—repeat measure design Aim To compare feasibility of electronic data collection data and symptom prevalence, frequency, severity, and distress from children with advanced cancer undergoing HSCT with a non-HSCT cohort.	Total number of participants 46 HSCT recipients *n* (%) 11 (23.91) Type of cancer *n* (%) Children who received HSCT: Hematologic malignancy: 5 (45) Brain Tumor: 1 (9) Solid Tumor: 5 (45) Demographics Age in years (mean + SD) Children who did not receive HSCT: 13.14 + 2.89 Children who received HSCT: 12.73 + 3.85 Sex—Male *n* (%) Children who did not receive HSCT: 21 (60) Children who received HSCT: 3 (27)	Abbreviated Pediatric Quality of Life and Memorial Symptom Assessment Scale (PQ-MSAS)
Ward, Smith [30] United States of America	Four regional paediatric transplantation and cellular therapy centers	Study design Longitudinal—repeat measures design Aim To determine the association between parent psychological distress and symptoms and HRQoL experienced by children and adolescents undergoing HSCT or CAR-T therapy.	Total number of participants 140 parent/child dyads 280 unique participants HSCT recipients *n* (%) Allogeneic: 81 (57.9) Autologous: 36 (25.7) CAR-T therapy: 23 (16.4) Type of cancer *n* (%) Malignancy: 102 (72.9) Red Blood Cell Disorder:23 (16.4) Immune deficiency: 11 (7.9) Metabolic disorder: 3 (2.1) Other: 1 (0.7) Demographics Age in years, mean (SD) 8.4 + 5 years Sex—Male *n* (%) 79 (56.4)	Memorial Symptom Assessment Scale (MSAS) Peds Quality of Life Cancer Module 3.0 (PedsQL CM)
Yildiz Kabak, Cetinkaya [39] Turkey	Two children’s hospitals; paediatric HSCT	Study design Randomized controlled trial Aim To examine the effects of multimodal exercise program on clinical status, emotional status, and QOL level in pediatric HSCT patients. In addition, to analyze QOL level of caregivers during HSCT period.	Total number of participants 26 HSCT recipients *n* (%) 26 (100) Type of cancer n (%) Leukaemia: 8 (30.8) Aplastic anemia: 5 (19.2) Thalassemia major: 5 (19.2) Immune deficiency: 2 (7.7) Other: 6 (23.1) Demographics Age in years, mean Intervention Group: 9.5 + 3.08 Control Group: 6.72 + 2.96 Sex—Male *n* (%) Not reported	The Wong–Baker FACES Pain Rating Scale Children’s Depression Inventory The Pediatric Quality of Life Inventory (PedsQL)

* Acute Myeloid Leukaemia (ALL) ** Acute Lymphocytic Leukaemia (ALL) *** Non-Hodgkin Lymphoma (NHL) **** Hodgkin Lymphoma (HL) ***** Myelodysplastic syndrome.

## Appendix C

**Table A3:** QuADS assessment of included studies.

Study	Theoretical or Conceptual Underpinning to the Research	Statement of Research Aim/s	Clear Description of Research Setting and Target Population	The Study Design Is Appropriate to Address the Stated Research Aim/s	Appropriate Sampling to Address the Research Aim/s	Rationale for Choice of Data Collection Tool/s	The Format and Content of Data Collection Tool Is Appropriate to Address the Stated Research Aim/s	Description of Data Collection Procedure	Recruitment Data Provided	Justification for Analytic Method Selected	The Method of Analysis Was Appropriate to Answer the Research Aim/s	Evidence That the Research Stakeholders Have Been Considered in Research Design or Conduct.	Strengths and Limitations Critically Discussed	QuADS * Total Score, n (%)
Bezinelli, Eduardo [26]	2	3	3	2	2	2	3	2	2	2	3	0	3	29 (74)
Cook, Vettese [34]	3	3	3	3	3	1	3	3	3	3	3	0	3	34 (87)
Dupuis, Johnston [3]	2	3	3	3	3	2	3	3	3	3	3	0	3	34 (87)
Dupuis, Johnston [35]	0	3	3	3	3	3	3	2	3	2	3	0	2	30 (77)
Ford, Vaughn [32]	1	3	2	3	2	0	3	1	1	3	3	0	0	22 (56)
Hetzer, Meryk [17]	0	2	3	3	1	2	3	3	1	0	3	0	2	23 (59)
Johnston, Gerbing [36]	0	3	3	3	3	3	3	2	3	0	3	0	2	28 (72)
Rodgers, Wills-Bagnato [29]	2	3	3	3	2	1	3	2	3	3	3	0	3	31 (79)
Rodgers, Highberger [18]	2	3	3	3	1	1	3	3	3	2	3	0	2	29 (74)
Sheikh, Miller [27]	1	1	3	3	0	3	3	1	3	0	3	1	2	24 (62)
Smith, Farhat [37]	1	1	3	3	2	1	3	2	3	1	3	0	3	26 (67)
Tomlinson, Tardif-Theriault [9]	0	3	3	3	3	2	3	2	3	2	3	0	2	29 (74)
Tomlinson, Dupuis [28]	0	3	3	3	3	2	3	3	3	3	3	0	3	32 (82)
Vettese, Cook [38]	3	3	3	3	3	3	3	3	3	3	3	2	2	37 (95)
Ward, Balian [31]	3	3	3	3	2	0	3	3	1	0	2	0	3	26 (67)
Ward, Smith [30]	3	3	3	3	3	1	3	2	3	3	3	0	3	33 (85)
Yildiz Kabak, Cetinkaya [39]	2	2	3	3	3	0	3	1	1	0	3	0	2	23 (59)

* Possible total score 0–39.
