# Supplementary material for: The Use of Patient-Reported Outcome Measures in Paediatric Haematopoietic Stem Cell Transplant: A Systematic Review
Source: Children (Basel). 2026 Mar 31;13(4):491. doi: 10.3390/children13040491 (PMC13114414; doi:10.3390/children13040491)
Supplement: Supplementary file 1 [file children-13-00491-s001.zip › children-4205804-supplementary.pdf]

Table S1. Characteristics of PROMs used in included studies

Study	PROM and type (generic, symptom specific)	Domains assessed	Validated	Method of collection (paper, electronic)	Frequency of assessment	Who completed the PROM (patient/ parent/ co-completion)	Intention of PROM (research, clinical care)	Who received the assessment information (for those PROMs used in clinical care)
Bezinelli et al. (2016)	**Patient-Reported Oral Mucositis Symptom (PROMS) scale**	**1.** Pain, measured by impact on:<br>• speaking<br>• eating and<br>• swallowing.	**Yes**	Not stated	**Four timepoints:**<br>*Time 1:* Prior to transplant<br>*Time 2:* 5 days after autologous or 8 days after allogeneic transplant<br>*Time 3:* at engraftment (absolute neutrophil count >500),<br>*Time 4:* 30 days after discharge	Parent proxy reported, patients aged 3 – 12 years;<br>Patient self-reported, patients >12 years.	**Research** - to measure effectiveness of intervention	Research team
	**Short form of the Oral Health Impact Profile (OHIP-14)**	**1.** Functional limitation<br>**2.** Physical pain<br>**3.** Psychological discomfort	**Yes** – for Brazilian and Portuguese participants	Not stated	**Four timepoints:**<br>*Time 1:* Prior to transplant<br>*Time 2:* 5 days after	Parent proxy reported, patients aged 3 – 12 years;	**Research** - to measure effectiveness of intervention	Research team

		**4.** Physical activity<br>**5.** Psychological disability<br>**6.** Social disability<br>**7.** Handicap or disability<br><br>14 questions			autologous or 8 days after allogeneic transplant<br>*Time 3:* at engraftment (absolute neutrophil count >500),<br>*Time 4:* 30 days after discharge	Patient self-reported, patients >12 years.		
Cook et al. (2020)	**Symptom Screening in Pediatrics Tool (SSPedi) via SPARK platform**	**1.** Physical<br>**2.** Psychological<br><br>**15 symptoms:** disappointed or sad, scared or worried, cranky or angry, problems thinking, body or face changes, tiredness, mouth sores, headache, other pain, tingling or numbness, throwing up, hunger changes, taste changes, constipation, and diarrhea	**Yes**	Electronic - iPad	**Daily for 5 consecutive days**	Patient self-reported.	**Research** - to measure feasibility of RCT.	SSPedi results were available to the child at any time and results from Day 1 -4 were shared with the bedside nurse, treating team, and also placed in the patients medical record
	**Symptom Screening in**	**1.** Physical<br>**2.** Psychological	**Yes**	Electronic - iPad	**Two timepoints:** *Day 1* and *Day*	Patient self-reported.	**Research** – to compare RCT intervention.	SSPedi results were

	**Pediatrics Tool (SSPedi)**	**15 symptoms:** disappointed or sad, scared or worried, cranky or angry, problems thinking, body or face changes, tiredness, mouth sores, headache, other pain, tingling or numbness, throwing up, hunger changes, taste changes, constipation, and diarrhea			*5* (baseline and final SSPedi score)			available to the child.
Dupuis et al. (2018)	**SSPedi**	**1.** Physical<br>**2.** Psychological<br><br>**15 symptoms:** disappointed or sad, scared or worried, cranky or angry, problems thinking, body or face changes, tiredness, mouth sores, headache, other pain, tingling or numbness, throwing up, hunger changes, taste changes,	**No**	Electronic	**Two timepoints** (more symptomatic group): *Day 1* and *Day 4*<br>**One timepoint:** (less symptomatic group): *Day 1*	Patient self-reported.	**Research** - to validate PROM and evaluate test-retest reliability and responsiveness	Research team

		constipation, and diarrhea						
	**Global Quality of Life (GQoL) visual categorical scale**	Quality of Life (QoL)	**Yes**	Electronic	Day 1	Self-report	**Research** – to perform construct validation	Research team
	**Children's International Mucositis Evaluation Scale (ChIMES)**	Oral mucositis	**Yes**	Electronic	Day 1	Self-report	**Research** – to perform construct validation	Research team
	**Faces Pain Scale - revised (FPS-R)**	Pain	**Yes**	Electronic	Day 1	Self-report	**Research** – to perform construct validation	Research team
	**Pediatric Nausea Assessment Tool (PeNAT)**	Nausea	**Yes**	Electronic	Day 1	Self-report	**Research** – to perform construct validation	Research team
	**5-point global symptom change scale**	Change in symptoms: Much worse, little worse, same, little better, and much better	**Yes**	Electronic	Day 4	Self-report	**Research** – to perform construct validation	Research team
Dupuis et al. (2025)	**SSPedi**	**1.** Physical<br>**2.** Psychological<br><br>**15 symptoms:**	**Yes**	Electronic	Intervention group<br>**Once daily for 5 consecutive days –**	Patient self-reported.	**Clinical care** - implementation of PROM	Intervention group: SSPedi results were available to the child at

		• feeling disappointed or sad<br>• feeling scared or worried<br>• feeling cranky or angry<br>• problems with thinking or remembering things<br>• changes in how your body or face look<br>• feeling tired<br>• mouth sores<br>• headache<br>• hurt or pain (other than headache)<br>• tingly or numb hands<br>• or feet<br>• throwing up or feeling like you may throw up<br>• feeling more or less hungry than you usually do<br>• changes in taste<br>• constipation, and<br>• diarrhea			Control group<br>**2 timepoints**<br>-Baseline and Day 5			any time and results from Day 1 -4 were shared with the bedside nurse, treating team, and also placed in the patients' health record. On Days 1 and 3, emails were sent to the responsible physician if any symptom was "a lot" or "extremely" bothersome. Reports and alerts contained links to clinical practice guidelines.

	**FPS-R**	Pain	**Yes**	Not stated	**2 timepoints -** Baseline and Day 5	Patient self-reported.	**Research** – to compare the mean difference in Day 5 scores between study groups.	Research team
	**Peds Quality of Life (PedsQL™) 3.0 Acute Cancer Module**	**1.** Pain and hurt<br>**2.** Nausea<br>**3.** Procedural anxiety<br>**4.** Treatment anxiety<br>**5.** Worry<br>**6.** Cognitive problems<br>**7.** Perceived physical appearance<br>**8.** Communication	**Yes**	Not stated	**2 timepoints -** Baseline and Day 5	Patient self-reported.	**Research** – to compare the mean difference in Day 5 scores between study groups.	Research team
Ford et al. (2024)	**mHealth application**	**1.** Symptom experience (occurrence and distress)<br>**2.** Mood (sliding scale)	Not reported	Electronic - Application	**Daily** for up to 120 study days.	Patient self-reported.	**Research** – to apply Network analysis and graphically represent relationships between symptoms, symptom distress, and mood.	Research team
Hetzer et al. (2023)	**ePROtect**	**1.** Pain<br>**2.** Nausea and appetite loss	Not reported	Electronic – touchscreen	**Daily** before 9:00 AM	Patient self-report or Parent	**Clinical Care** – to implement ePROtect	ePROtect scores were reviewed by

		**3.** Physical functioning<br>**4.** Sleep disturbance		smartphone or tablet	throughout the treat-<br>ment period	proxy-report		the treating team before the morning visit (7 days a week). Any score deviations were discussed immediately and interventions initiated as per the standard operating procedure.
Johnston et al. (2015)	**PedsQL™ 4.0 Generic Core Scales**	**1.** Physical functioning<br>**2.** Emotional functioning<br>**3.** Social functioning<br>**4.** School functioning	**Yes**	Paper	**Eight timepoints**<br>• Pre and post treatment<br>• Pre and post transplant<br>Up to 3 years post diagnosis	Parent-proxy reported or Patient self-reported	**Research** – to evaluate the impact of a patient-reported outcome (PRO) coordinator on completion rates	Research team
	**Peds Quality of Life (PedsQL™) 3.0 Acute Cancer Module**	**1.** Pain and hurt<br>**2.** Nausea<br>**3.** Procedural anxiety<br>**4.** Treatment anxiety<br>**5.** Worry	**Yes**	Paper	**Eight timepoints**<br>• Pre and post treatment<br>• Pre and post transplant	Parent-proxy reported or Patient self-reported	**Research** – to evaluate the impact of a patient-reported outcome (PRO) coordinator on	Research team

		**6.** Cognitive problems<br>**7.** Perceived physical appearance<br>**8.** Communication			Up to 3 years post diagnosis		completion rates	
	**PedsQL Mulitdimensional Fatigue Scale**	**1.** General fatigue,<br>**2.** Sleep/ rest fatigue and<br>**3.** Cognitive fatigue.	**Yes**	Paper	**Eight timepoints**<br>• Pre and post treatment<br>• Pre and post transplant<br>Up to 3 years post diagnosis	Parent-proxy reported or Patient self-reported	**Research** – to evaluate the impact of a patient-reported outcome (PRO) coordinator on completion rates	Research team
Rodgers et al. (2015)	**Memorial Symptom Assessment Scale (MSAS)**	**1.** Physical symptoms<br>• Tired<br>• Sad<br>• Itchy<br>• Worry<br>• Pain<br>• Difficulty eating<br>• Vomiting<br>• Insomnia<br><br>**2.** Global distress	**Yes**	Not stated	**6 time points** –<br>• Monthly, within 7 days of their 1st through 6th month post-HSCT.	Patient self-reported	**Research** – to estimate the association between health-related quality of life (HRQoL) and individual symptom presence at 1 month post allogeneic transplant	Research team
	**PedsQL CM™**	**1.** Pain and hurt<br>**2.** Nausea<br>**3.** Procedural anxiety	**Yes**	Not stated	**6 time points** – Monthly, within 7 days of their 1st – 6th month post-	Patient self-reported	**Research** – to describe HRQoL changes during the first 6	Research team

		**4.** Treatment anxiety<br>**5.** Worry<br>**6.** Cognitive problems<br>**7.** Perceived physical appearance<br>**8.** Communication					months post allogeneic transplant.	
Rodgers et al. (2019)	**MSAS 10-18 years (MSAS 10-18)**	**1.** Physical symptoms (12)<br>**2.** Psychological symptoms (6)<br>**3.** Global distress	**Yes**	Paper	**4 timepoints** Prior to transplant admission and then every 30 days for 3 additional time points HSCT.	Patient self-reported	**Research** – to describe symptom trajectories from pre-transplant through 90 days post-transplant	Research team
Sheikh et al. (2021)	**MD Anderson Symptom Inventory – adolescent (MDASI- Adol)**	**1.** Physical symptoms (13)<br>• Pain<br>• Fatigue<br>• Nausea<br>• Disturbed sleep<br>• Feeling disturbed or upset<br>• Shortness of breath<br>• Trouble remembering things<br>• lack of appetite,	**Yes**	Paper	**Pre transplant and weekly during hospitalisation**.	Patient self-reported	**Research** – to determine symptom burden around time of transplant and to describe the clinical utility of implementing a symptom screening tool	Results uploaded to the electronic medical record

		• Feeling drowsy<br>• Dry mouth<br>• Feeling sad<br>• Vomiting<br>• Numbness or tingling<br><br>**2.** QoL (6)<br>• General activity<br>• Mood<br>• Work<br>• Relationships<br>• Walking<br>• Enjoyment of life						
Smith et al. (2022)	**Patient Reported Outcomes Measurement Information System (PROMIS)**	**1.** Anxiety<br>**2.** Depressive symptoms<br>**3.** Pain interference<br>**4.** Fatigue<br>**5.** Physical function-mobility<br>**6.** Peer relationships	**Yes**	Not reported	**3 timepoints –**<br>• Baseline - admission for transplant<br>• Day of hospital discharge<br>6 weeks after discharge.	Patient self-reported or Parent proxy-reported.	**Research –** to measure the outcomes of exercise program on QoL.	Research team
Tomlinson, Tardif-Theriault, et al. (2024)	**Co-SSPedi**	**1.** Physical<br>**2.** Psychological<br><br>**15 symptoms:**<br>• feeling disappointed or sad<br>• feeling scared or worried	**Yes**	Electronic - iPad via SPARK platform	**Once only**	Co-completion - by child and guardian together	**Research** - to compare results and variability of different SSPedi collection formats	Research team

		• feeling cranky or angry<br>• problems with thinking or remembering things<br>• changes in how your body or face look<br>• feeling tired<br>• mouth sores<br>• headache<br>• hurt or pain (other than headache)<br>• tingly or numb hands<br>• or feet<br>• throwing up or feeling like you may throw up<br>• feeling more or less hungry than you usually do<br>• changes in taste<br>• constipation, and<br>• diarrhea						
	**Proxy SSPedi**	As above	**Yes**	Electronic - iPad via SPARK platform	**Once only**	Parent proxy-reported	**Research** - to compare results and variability of different SSPedi	Research team

							collection formats	
	**Self-reported SSPedi**	As above	**Yes**	Electronic - iPad via SPARK platform	**Once only**	Patient self-reported	**Research** - to compare results and variability of different SSPedi collection formats	Research team
Tomlinson, Dupuis, et al. (2024)	**Co-Symptom Screening in Pediatrics Tool (Co-SSPedi)**	**1.** Physical<br>**2.** Psychological<br><br>**15 symptoms:**<br>• feeling disappointed or sad<br>• feeling scared or worried<br>• feeling cranky or angry<br>• problems with thinking or remembering things<br>• changes in how your body or face look<br>• feeling tired<br>• mouth sores<br>• headache<br>• hurt or pain (other than headache)	**No**	Electronic - iPad via SPARK platform	**Two timepoints** (more symptomatic group): *Day 1* and *Day 4*<br>**One timepoint:** (less symptomatic group): *Day 1*	Parent/ child dyad co-completion	**Research –** to validate Co-SSpedi	Research team

		• tingly or numb hands<br>• or feet<br>• throwing up or feeling like you may throw up<br>• feeling more or less hungry than you usually do<br>• changes in taste<br>• constipation, and<br>diarrhea						
	**Global Quality of Life (GQoL) visual categorical scale**	Quality of Life (QoL)	**Yes**	Electronic	**Day 1**	Parent proxy-reported	**Research** – to perform construct validation	Research team
	**Children's International Mucositis Evaluation Scale (ChIMES)**	Oral mucositis	**Yes**	Electronic	**Day 1**	Parent proxy-reported	**Research** – to perform construct validation	Research team
	**Faces Pain Scale - revised (FPS-R)**	Pain	**Yes**	Electronic	**Day 1**	Parent proxy-reported	**Research** – to perform construct validation	Research team
	**Pediatric Nausea Assessment Tool (PeNAT)**	Nausea	**Yes**	Electronic	**Day 1**	Parent proxy-reported	**Research** – to perform construct validation	Research team
	**5-point global symptom change scale**	Change in symptoms: Much worse, little worse,	**Yes**	Electronic	**Day 4**	Parent/ child dyad	**Research** – to perform	Research team

		same, little better, and much better				co-completion	construct validation	
Vettese et al. (2019)	**SSPedi**	**1.** Physical<br>**2.** Psychological<br><br>**15 symptoms:**<br>• feeling disappointed or sad<br>• feeling scared or worried<br>• feeling cranky or angry<br>• problems with thinking or remembering things<br>• changes in how your body or face look<br>• feeling tired<br>• mouth sores<br>• headache<br>• hurt or pain (other than headache)<br>• tingly or numb hands<br>• or feet<br>• throwing up or feeling like you may throw up	**Yes**	Electronic - iPad	**Daily for 5 consecutive days**	Patient self-reported	**Research** – to determine feasibility of longitudinal utilization of SSPedi	Research team

		• feeling more or less hungry than you usually do<br>• changes in taste<br>• constipation, and<br>diarrhea						
Ward et al. (2020)	**The Pediatric Quality of Life and evaluation of Symptoms study (PQ-MSAS)**	**1.** Physical<br>**2.** Psychological<br><br>7 – 12 years version<br>**8 symptoms:**<br>• pain<br>• fatigue<br>• sadness<br>• worry<br>• appetite<br>• nausea<br>• sleeping difficulties<br>• shortness of breath<br><br>13 – 18 years version<br>**11 symptoms:**<br>• pain<br>• fatigue<br>• sadness<br>• worry<br>• appetite<br>• nausea	**No**	Electronic	Every two weeks for up to 61 weeks.	Patient self-report	**Research** – to evaluate feasibility and acceptability of prospective electronic data collection	Research team

		• sleeping difficulties<br>• shortness of breath<br>• diarrhoea<br>• constipation<br>• irritability						
Ward et al. (2023)	**Memorial Symptom Assessment Scale (MSAS)**	1. Physical<br>2. Psychological	**Yes**	Electronic or Paper	**4 time points:**<br>• prior to cell infusion<br>• day + 30<br>• day + 60<br>• day + 90	Patient self-reported<br><br>Parent proxy-report for younger children	**Research** – to measure relationships between patient symptoms and HRQoL and parent psychological distress	Research team
	**Peds Quality of Life Cancer Module 3.0 (PedsQL CM)**	1. Physical<br>2. Psychological<br><br>**8 dimensions:**<br>• pain<br>• nausea<br>• procedural anxiety<br>• treatment anxiety<br>• worry<br>• cognitive problems<br>• perceived physical appearance	**Yes**	Electronic or Paper	**4 time points:**<br>• prior to cell infusion<br>• day + 30<br>• day + 60<br>• day + 90	Patient self-reported<br><br>Parent proxy-report for younger children	**Research** – to measure relationships between patient symptoms and HRQoL and parent psychological distress	Research team

		• communication.						
Yildiz Kabak et al. (2019)	**The Wong-Baker FACES Pain Rating Scale**	**1.** Pain<br>**2.** Fatigue	**Yes**	Not stated	**3 timepoints:**<br>• Baseline on admission<br>• At discharge<br>• One month post discharge	Patient self-reported	**Research** – to measure outcomes of the study intervention	Research team
	**Children's Depression Inventory**	**1.** Depression<br>27 items, symptom oriented	**Yes**	Not stated	**3 timepoints:**<br>• Baseline on admission<br>• At discharge<br>• One month post discharge	Patient self-reported	**Research** – to measure outcomes of the study intervention	Research team
	**The Pediatric Quality of Life Inventory (PedsQL) Cancer Module**	**1.** Pain and hurt<br>**2.** Nausea<br>**3.** Procedural anxiety<br>**4.** Treatment anxiety<br>**5.** Worry<br>**6.** Cognitive problems<br>**7.** Perceived physical appearance<br>**8.** Communication.	**Yes**	Not stated	**3 timepoints:**<br>• Baseline on admission<br>• At discharge<br>• One month post discharge	Patient self-reported	**Research** – to measure outcomes of the study intervention	Research team
